# FeO*_x_*‐Based Materials for Electrochemical Energy Storage

**DOI:** 10.1002/advs.201700986

**Published:** 2018-04-23

**Authors:** Jingyi Ma, Xiaotian Guo, Yan Yan, Huaiguo Xue, Huan Pang

**Affiliations:** ^1^ School of Chemistry and Chemical Engineering Institute for Innovative Materials and Energy Yangzhou University Yangzhou 225009 Jiangsu P. R. China

**Keywords:** batteries, electrochemical energy storage, FeO*_x_*‐based materials, supercapacitors

## Abstract

Iron oxides (FeO*_x_*), such as Fe_2_O_3_ and Fe_3_O_4_ materials, have attracted much attention because of their rich abundance, low cost, and environmental friendliness. However, FeO*_x_*, which is similar to most transition metal oxides, possesses a poor rate capability and cycling life. Thus, FeO*_x_*‐based materials consisting of FeO*_x_*, carbon, and metal‐based materials have been widely explored. This article mainly discusses FeO*_x_*‐based materials (Fe_2_O_3_ and Fe_3_O_4_) for electrochemical energy storage applications, including supercapacitors and rechargeable batteries (e.g., lithium‐ion batteries and sodium‐ion batteries). Furthermore, future perspectives and challenges of FeO*_x_*‐based materials for electrochemical energy storage are briefly discussed.

## 1 Introduction

Currently, with the rapid development of the economy, the overconsumption of fossil fuels has resulted in great demand for energy. As a consequence, a sustainable and low‐cost way to store energy more efficiently has been continuously explored in recent years, especially for studies on electrochemical energy storage. Green electrochemical energy storage devices mainly include supercapacitors (SCs)^1^, ^2^ and rechargeable batteries^3^ (lithium‐ion batteries (LIBs),^4^ sodium‐ion batteries (SIBs), lithium–sodium ion batteries (LSBs), and so on).^5^, ^6^

Iron (Fe) is the fourth richest element on earth, forming much of Earth's outer and inner core.^7^ FeO*_x_* materials, including FeO, Fe_2_O_3_, and Fe_3_O_4_, are extensively used in industrial production for products used in daily life.^8^, ^9^, ^10^, ^11^ Due to their high theoretical capacity of 800–1000 mA h g^−1^, Fe_2_O_3_ and Fe_3_O_4_ have received much attention. In addition, FeO*_x_*‐based materials, other metal oxide materials, and carbon materials have been widely reported for applications in electrochemical energy storage,^12^, ^13^, ^14^, ^15^ which can effectively reduce the obvious volume change that results in capacity decay and poor performance.^16^

**Figure 1:**
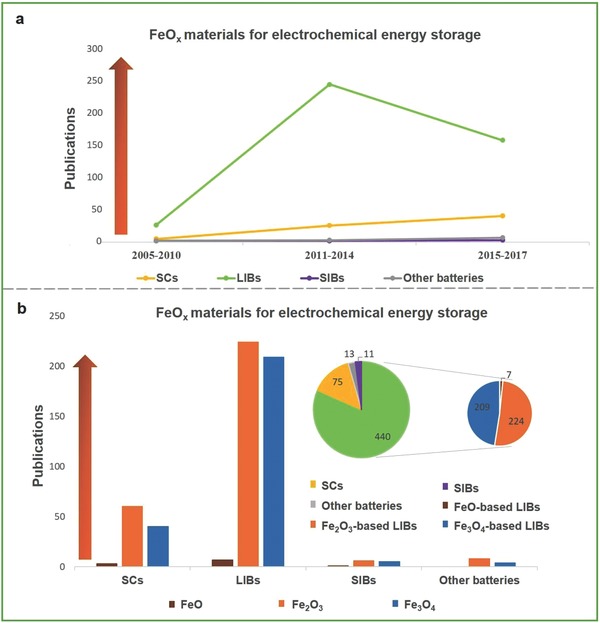
a) Line chart of the research trend of FeO*_x_*‐based materials for supercapacitors, lithium ion battery, sodium ion battery, and other batteries in recent years. b) Bar chart and pie chart of the ratio of FeO*_x_*‐based materials applied in electrochemical energy storage (others containing lithium–sodium ion battery, alkaline secondary battery, and Fe–air battery). Source for data: Web of Science (2008–2017).

Considerable work of nanostructured Fe‐based and FeO*_x_*‐based materials has been reported.^17^, ^18^, ^19^ Fe‐based materials including Fe_2_O_3_, Fe_3_O_4_, FeOOH, FeO*_x_*, CoFe_2_O_4_, and MnFe_2_O_4_ were investigated by Zeng et al. when applied as electrodes for SCs.^1^ Zhang et al. reviewed FeO*_x_*‐based materials (Fe_2_O_3_, Fe_3_O_4_) in LIBs based on 1D nanowires (NWs)/rods, 2D nanosheets/flakes, 3D porous/hierarchical architectures, various hollow structures, and hybrid nanostructures of FeO*_x_* and carbon (including amorphous carbon, carbon nanotubes, and graphene), the nanostructures and electrochemical performance of which are also presented.^20^ The morphology, composition, porosity, and surface characteristics all affect the performance of FeO*_x_*‐based materials.^15^ Literature progress of FeO*_x_*‐based materials for SCs, LIBs, SIBs, and other batteries has been demonstrated, as shown in **Figure** **1**a,b. Therefore, it is necessary to provide a review of FeO*_x_*‐based materials for applications in electrochemical energy storage.

In this review, we focus on the FeO*_x_*‐based materials for applications in electrochemical energy storage, including SCs and rechargeable batteries (LIBs, SIBs, LSBs, and so on). The comparison of FeO*_x_*‐based materials is on **Table** **1**. Generally, Fe_2_O_3_ and Fe_3_O_4_ have been combined with metal‐based materials and carbon materials, such as carbon nanotubes (CNTs) and graphene. In addition, their synthesis, structure, and electrochemical performance are discussed as well. Finally, future perspectives and challenges of FeO*_x_*‐based materials for electrochemical energy storage are briefly presented.

**Table 1:** The comparison of FeO*_x_*‐based materials for applications in energy storage

Materials	FeO‐based materials	Fe_2_O_3_‐based materials	Fe_3_O_4_‐based materials
Synthesis	Hard to obtain, unstable	Easier to obtain than others (hydrothermal method)	Complicated synthetic process
Morphology	Nanoparticles, nanowires	Nanoparticles, nanospindles, microspheres, nanowires, nanorods nanocubes, films, and so on	Nanoparticles, nanospindles, nanofibers, films, nanospheres, nanowires, nanorods, and so on
**Performance in LIBs**			
Voltage range	0–3 V	0–3 V	0–3 V
Reversible capacity	600–800 mA h g^‐1^, 0.1–1 A g^‐1^	600–1000 mA h g^‐1^, 0.5–1 A g^‐1^	900–1500 mA h g^‐1^, 1–10 A g^‐1^
Cycle ability	95–100%, 50–100 cycles	55–99%, 50–500 cycles	50–95%, 50–200 cycles
Rate capability	500–600 mA h g^‐1^, 0.5–1 A g^‐1^	900–1300 mA h g^‐1^, 0.1–1 A g^‐1^	900–1400 mA h g^‐1^, 1–9 A g^‐1^
**Performance in SCs**			
Specific capacitance	500–1000 F g^‐1^, 1–2 A g^‐1^	150–300 F g^‐1^, 1–10 A g^‐1^	100–200 F g^‐1^, 0.5–10 A g^‐1^
Energy density	20–50 Wh kg^‐1^	40–80 Wh kg^‐1^	30–60 Wh kg^‐1^
Suitable applications	SCs	LIBs	LIBs

## 2 Crystal Structures and Charge Storage Mechanism

To explore better performances for the applications in electrochemical energy storage, the charge storage mechanism and the effect of crystal structures on the electrochemical performances should be studied.

**Figure 2:**
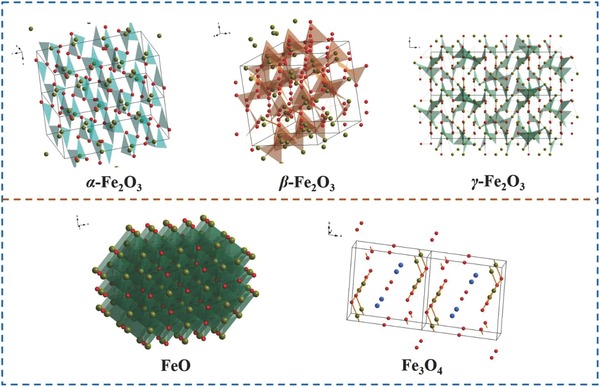
Crystal structures of α‐, β‐, γ‐Fe_2_O_3_, FeO, and Fe_3_O_4_ (yellow globule: Fe^2+^/Fe^3^. Red globule: Fe^3+^).

**Figure 3:**
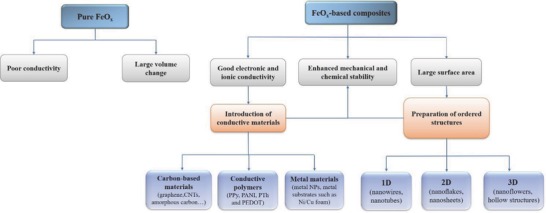
The shemetic illustration of pure FeO*_x_* and FeO*_x_*‐based composites including the factors of electrochemical performances.

On the basis of the charge storage mechanisms, SCs can be divided into electrical double‐layer capacitors (EDLCs) and pseudocapacitors. The charge storages of them all occur at the surface or in the thin layer parts of active materials. The former focuses on charge separation and accumulation, while the latter are based on reversible and fast redox reactions. Pseudocapacitors have attracted more attention owing to the higher energy density compared to that of EDLCs. On the one hand, FeO*_x_* are widely used in pseudocapacitors due to the various valence states (Fe^0^, Fe^2+^, Fe^3+^, and so on) and unique crystal structures of their ions, which ensure the occurrence of reversible and fast redox reactions. Fe_2_O_3_ generally has three crystal structures, including α‐Fe_2_O_3_, β‐Fe_2_O_3_, γ‐Fe_2_O_3_. β‐Fe_2_O_3_ and γ‐Fe_2_O_3_ are the transition states for the evolution to α‐Fe_2_O_3_. Meanwhile, the crystal structure of α‐Fe_2_O_3_ is more stable than that of β‐Fe_2_O_3_ and γ‐Fe_2_O_3_, which ensures the long life for SCs (**Figure** **2**).^1^ In addition, more conductive materials, including carbon‐based materials, conductive polymer, and metal materials, can be combined with FeO*_x_* in order to improve the conductivity (**Figure** **3**). Besides, desirable structures are designed to increase the surface area and improve the stability.

Meanwhile, the charge mechanism of rechargeable batteries is related with fast transfer of Li^+^. It is crucial to shorten the path length for transport of Li^+^ and ensure the absorption and storage of large amount of Li^+^ without causing deterioration. The theoretical capacitances of FeO, α‐Fe_2_O_3_, and Fe_3_O_4_ are about 600, 1000, and 800 mA h g^−1^, respectively. In addition, better conductivity and more desirable morphologies have a good influence on the transition, absorption, and storage of large amount of lithium ions without causing deterioration. The transfer of Li^+^ can occur on the surface and in most of the active materials. Take LIBs as an example, the electrochemical reactions of FeO*_x_* are presented, respectively

(1) $$\begin{array}{l} \mathrm{FeO}:\;\mathrm{FeO}+x{\mathrm{Li}}^{+}+xe^{-}\to {\mathrm{Li}}_{x}\mathrm{FeO};\;\\ \;\;\;\;\;{\mathrm{Li}}_{x}\mathrm{FeO}+\left(2-x\right)\;{\mathrm{Li}}^{+}+\left(2-x\right)e^{-}\to {\mathrm{Li}}_{2}O+\mathrm{Fe} \end{array}$$

(2) $${\mathrm{Fe}}_{2}O_{3}:\;{\mathrm{Fe}}_{2}O_{3}+6{\mathrm{Li}}^{+}+{\mathrm{6e}}^{-}\to 2\mathrm{FeO}+{\mathrm{3Li}}_{2}O$$

(3) $$\begin{array}{l} {\mathrm{Fe}}_{3}O_{4}:\;{\mathrm{Fe}}_{3}O_{4}+x{\mathrm{Li}}^{+}+xe^{-}\to {\mathrm{Li}}_{x}{\mathrm{Fe}}_{3}O_{4};\;\\ {\mathrm{Li}}_{x}{\mathrm{Fe}}_{3}O_{4}+\left(8-x\right){\mathrm{Li}}^{+}+\left(8-x\right)e^{-}\to 4{\mathrm{Li}}_{2}O+3\mathrm{Fe} \end{array}$$

Generally, major efforts have been made, as shown in Figure 3, in order to achieve enhanced performances not only in SCs but also in LIBs. The problems of poor conductivity and large volume change can be largely diminished so that the FeO*_x_*‐based materials with enhanced stability will be examined as promising anode electrode materials in rechargeable batteries, especially high performance LIBs.

## 3 Fe_2_O_3_‐Based Nanomaterials

In the modern society, Fe_2_O_3_ materials play an important role in electrochemical energy storage systems.^21^ Due to its abundance, environmental friendliness, good electrochemical activity, high stability under ambient conditions, and low cost, Fe_2_O_3_ has attracted much attention as a negative electrode material in electrochemical energy storage.

### 3.1 Supercapacitors

According to the energy storage mechanisms, SCs can be divided into EDLCs, pseudocapacitors, and hybrid capacitors. Moreover, the electrochemical performance of Fe_2_O_3_‐based materials for SCs depends mainly on the structure and test conditions, including the electrolyte, applied mass loading voltage and electrode configuration, or device assembly. Here, we focus on the material selection, synthesis, structure, and electrochemical performance.^22^, ^23^, ^24^ Currently, FeO*_x_*, along with metals, other MO*_x_*, and carbon materials, have been studied and widely reported. Exploring novel FeO*_x_*‐based materials with desirable structures is an effective way to further improve the electrochemical performance of SCs.^25^, ^26^, ^27^

#### 3.1.1 Pure Fe_2_O_3_

Pure Fe_2_O_3_ nanoparticles (NPs),^28^ films,^29^ nanosheets,^30^ and hollow nanoshuttles^31^ have been widely explored for applications in SCs.

**Figure 4:**
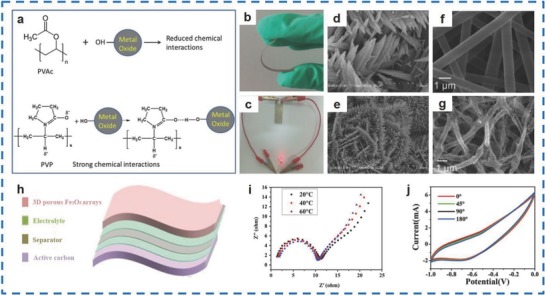
a) A schematic demonstrating the polymer PVP and PVAc–MO interactions. b) A photograph showing excellent flexibility. c) A photograph of a red LED powered by the flexible SCs. d,e) Porous α‐Fe_2_O_3_ after annealing at 400 °C in air for 4 h. f,g) SEM images of the Fe(acac)_3_‐PVP electrospun fibers and the corresponding sintered PFs. h) A schematic diagram of the fiber‐based all‐solid‐state flexible asymmetric SC. i) Nyquist plots of α‐Fe_2_O_3_ hollow nanoshuttles at different temperatures. j) Cyclic voltammetry curves (CVs) of the flexible PSI SC at 0^o^, 45^o^, 90^o^, and 180^o^. (a,f,g) Reproduced with permission.^32^ Copyright 2013, Royal Society of Chemistry; (b–e,h,j) Reproduced with permission.^33^ Copyright 2015, Royal Society of Chemistry; (i) Reproduced with permission.^31^ Copyright 2016, Elsevier.

Generally, strategies for the synthesis of pure Fe_2_O_3_ mainly include the electrospinning,^32^ template,^33^ and hydrothermal methods.^31^ Binitha et al. fabricated two different morphologies of α‐Fe_2_O_3_, nanograin (NG), and porous fiber (PF), using the electrospinning method to obtain α‐Fe_2_O_3_ particles of 21 and 53 nm in size, respectively.^32^ Ferric acetyl acetonate (Fe(acac)_3_), which was used as an α‐Fe_2_O_3_ precursor together with Fe_2_O_3_‐polyvinyl pyrrolidone (PVP)/polyvinyl acetate (PVAc) fibers, was used for the synthesis of α‐Fe_2_O_3_ PFs and NGs (**Figure** **4**f,g). In addition, the evolution of the structure contributed to the chemical interactions between the Fe(acac)_3_ and polymer, as shown in Figure 4a. The α‐Fe_2_O_3_ PFs delivered a specific capacitance and a power density of 348 F g^−1^ and 1149 W kg^−1^ at 5 A g^−1^, respectively, which were higher than those of the α‐Fe_2_O_3_ NGs (159 F g^−1^ and 997 W kg^−1^). In addition, the as‐obtained materials also exhibited superb cycling performance (256 F g^−1^ at 1 mV s^−1^). Additionally, flexible SCs were obtained by Nan et al. by fabricating porous spinous Fe_2_O_3_ materials (PSI) on a thin Fe substrate with superior flexibility (Figure 4b) via a template method.^33^ Figure 4d,e shows the as‐prepared products with 20–50 nm in width and 200–400 nm in length after heat treating at 400 °C for 4 h. It was demonstrated that the as‐prepared SCs maintained a good current at different curvatures due to the flexibility of the Fe substrate (Figure 4j). As a result, PSI displayed specific capacitances of 524.6, 362.5, and 313.1 F g^−1^ at 1, 10, and 20 A g^−1^, respectively, and showed superior cycling stability (92.9% of the initial value) over 5000 cycles. In conclusion, the fiber‐based all‐solid‐state flexible SCs (Figure 4h) successfully illuminated an light emitting diode (LED) (Figure 4c). Furthermore, a hydrothermal method was used by Zheng et al. for the fabrication of α‐Fe_2_O_3_ hollow nanoshuttles with a uniform wall thickness of 30 nm and a length of 100 nm.^31^ Figure 4i shows that the charge transfer resistances of 4.42, 4.12, and 3.80 Ω were achieved at 20, 40, and 60 °C, respectively, and the α‐Fe_2_O_3_ hollow nanoshuttles displayed an excellent capacitance of 249 F g^−1^ at 0.5 A g^−1^.

#### 3.1.2 Fe_2_O_3_/Carbon Nanomaterials

Fe_2_O_3_ combined with carbon materials, such as CNTs and graphene, have been reported as anodes for application in SCs. The porous α‐Fe_2_O_3_/CNTs hierarchical nanostructure^34^ and Fe_2_O_3_/multiwall carbon nanotubes (MWCNTs) thin films^35^, ^36^ have been studied. The α‐Fe_2_O_3_/MWCNTs materials synthesized by Zhao et al. via a scalable spray deposition method displayed a high power density of 50 W h kg^−1^ for hybrid SCs at 1000 W kg^−1^.^36^ Nanoporous Fe_2_O_3_ with 5 wt% CNT materials, which were prepared by Xu et al. using scanning‐mode N_2_ atmospheric pressure plasma jets, displayed a specific capacitance of 54 F g^−1^ at 2 mV s^−1^.^37^ Cheng et al. coated Fe_2_O_3_ nanohorns with a conductive CNT network using the chemical vapor deposition (CVD) method.^34^ The as‐fabricated materials delivered a maximum capacitance of 296.3 F g^−1^ at 5 mV s^−1^ and remained at 80% and 60% of the initial capacitance over 200 and 1000 cycles, respectively. Furthermore, a specific capacitance of 296.3 F g^−1^ was obtained by the Fe_2_O_3_/CNTs sponge over 1000 cycles of compression under 50% strain, demonstrating great potential in flexible energy storage devices.

In addition to Fe_2_O_3_/CNTs composites, Fe_2_O_3_/graphene composites, such as Fe_2_O_3_/graphene aerogel (GA) composites^38^, ^39^ and Fe_2_O_3_ nanoplates/graphene,^40^ have been explored mainly through hydrothermal/solvothermal method. The Fe_2_O_3_ NPs with the sizes of 30–60 nm encapsulated in GA were synthesized by Song et al., which displayed a specific capacitance of 81.3 F g^−1^ at 1 A g^−1^.^39^ Similarly, Fe_2_O_3_/GA materials were also fabricated by Khattak et al., and they delivered a specific capacitance of 440 F g^−1^ at 0.45 A g^−1^.^38^ Furthermore, the as‐obtained materials maintained 90% of their initial capacitance, even after 2200 cycles. Apart from the Fe_2_O_3_/GA materials, α‐Fe_2_O_3_ nanoplates connected with reduced graphene oxide (rGO) network materials were prepared by Quan et al. using a hydrothermal method.^41^ A specific capacitance of 903 F g^−1^ at 1 A g^−1^ was achieved for the α‐Fe_2_O_3_/rGO materials, which was higher compared to that of pure Fe_2_O_3_ (347 F g^−1^).

Furthermore, Fe_2_O_3_ NP clusters/rGO was synthesized by Hu et al. via gel formation reaction, hydrothermal process, vacuum filtration, and electrochemical reduction four procedures for applications in flexible asymmetric supercapacitors, which are of great potential for electrochemical energy storage owing to the superior energy density.^42^ The rGO sheets ensured the superior conductivity and flexibility while the Fe_2_O_3_ NP clusters had satisfactory performances for pseudocapacitors without weakening the high conductivity of the overall hybrid paper so that a high energy density of 178.3 F cm^−3^ is obtained at the 1 mV s^−1^. Guan et al. synthesized the graphite foam (GF)/CNT/Fe_2_O_3_ (graphite foam carbon/nanotube framework) by coating GF/CNT substrate with nanocrystalline Fe_2_O_3_ which was deposited with the use of atomic layer deposition (ALD).^43^, ^44^ It was demonstrated that a high energy of ≈74.7 Wh kg^−1^ at a ≈1400 W kg^−1^ and a capacitance retention of ≈95.4% over 50 000 cycling tests were achieved.

#### 3.1.3 Fe_2_O_3_/Metal‐Based Nanomaterials

Fe_2_O_3_‐MO*_x_* materials, such as CeO_2_/Fe_2_O_3_ composite nanospindles (CNSs),^45^ V_2_O_5_/α‐Fe_2_O_3_ nanotubes,^46^ Fe_2_O_3_/CuO thin films,^47^ NiO nanosheets/Fe_2_O_3_ nanorods (NRs),^48^ and RuO_2_/Fe_2_O_3_ NPs,^49^ have also been applied in the SCs.

Arul et al. fabricated CNSs with a crystallite size of 4.47 nm, and the size was the smallest among CeO_2_ NPs (15.75 nm) and Fe_2_O_3_ NRs (4.62 nm) using a coprecipitation method.^45^ In addition, a specific capacitance of 142.6 F g^−1^ at 5 mV s^−1^ and an outstanding capacitance retention of 94.8% at the 1000th cycle were achieved, demonstrating great potential for applications in SCs. Additionally, V_2_O_5_‐decorated α‐Fe_2_O_3_ nanotubes were fabricated by Nie et al. using an electrospinning approach.^46^ The V_2_O_5_/Fe_2_O_3_ materials with weight ratios of 0%, 1.0%, 2.5%, 5.0%, and 10.0% were named VFNT0, VFNT1, VFNT2, VFNT3, and VFNT4, respectively. The VFNT1 materials with a diameter of 117 nm delivered a specific capacitance of 183 F g^−1^ at 1 A g^−1^, which was higher than that of VFNT0 (100.5 F g^−1^). In addition, the materials achieved a retention of more than 60% even at 5 A g^−1^. Furthermore, Jiao et al. fabricated α‐Fe_2_O_3_ NRs/NiO nanosheets 30 nm in diameter and 300 nm in length via a hydrothermal method, and they displayed a superb areal capacitance of 557 mF cm^−2^.^50^ Moreover, a specific capacitance retention of ≈96.2% was obtained over 3000 cycles at 1 mA cm^−2^, providing great potential for applications in SCs.

### 3.2 LIBs

Motivated by the demand for portable electronic devices and electric vehicles, rechargeable LIBs have been developed over the last few decades and have attracted increasing attention. Currently, they demonstrate barriers to realization using commercial graphite anodes because of the low lithium‐ion storage capacity (372 mA h g^−1^). To improve the energy density of batteries, various promising materials with high theoretical capacities have been used. In addition, many significant studies have been conducted in recent decades to develop the electrochemical energy storage of LIBs.^51^, ^52^, ^53^, ^54^, ^55^

#### 3.2.1 Pure Fe_2_O_3_

**Figure 5:**
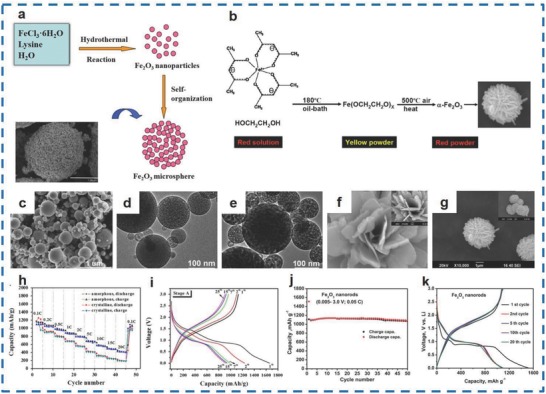
a) Schematic for the formation mechanism of Fe_2_O_3_ microspheres. b) Overall synthesis procedure of α‐Fe_2_O_3_ clusters. c) SEM images of γ‐Fe_2_O_3_. d,e) TEM images of α‐Fe_2_O_3_ and γ‐Fe_2_O_3_. f,g) SEM images of the prepared α‐Fe_2_O_3_ powders. h) Comparison of rate capabilities of amorphous and crystalline Fe_2_O_3_ anode. i) The Fe_2_O_3_/Li half‐cells were cycled at 100 mA h g^−1^ (≈0.1 C) for the first 5 cycles and then 1000 mA h g^−1^ (≈1 C) for the following cycles. Charge/discharge curves of amorphous Fe_2_O_3_ anode for stage A. j) Capacity versus cycle number plot of Fe_2_O_3_ at 50 mA g^−1^ (0.05 C). k) Voltage versus capacity profiles of α‐Fe_2_O_3_ at 50 mA g^−1^ (0.05 C). (a) Reproduced with permission.^58^ Copyright 2013, Elsevier; (b,f,g) Reproduced with permission.^59^ Copyright 2013, Elsevier; (c–e) Reproduced with permission.^56^ Copyright 2014, Elsevier; (h,i) Reproduced with permission.^60^ Copyright 2014, Elsevier; (j,k) Reproduced with permission.^57^ Copyright 2012, Royal Society of Chemistry.

Pure Fe_2_O_3_ is mainly obtained by the spray pyrolysis method,^56^ electrospinning‐annealing method,^57^ hydrothermal method,^58^ and thermal decomposition method.^59^ Xu et al. synthesized γ‐Fe_2_O_3_ with a crystalline structure of ≈1 µm in diameter and a pore size of 0.4 µm (**Figure** **5**c–e) through aerosol spray pyrolysis at different temperatures.^56^ The γ‐Fe_2_O_3_ spherical particles exhibited a specific capacity of 800 mA h g^−1^ at 0.5 C after 300 cycles and remained at 300 mA h g^−1^ at 10 C. Jiang et al. synthesized hierarchically porous Fe_2_O_3_, which exhibited initial discharge/charge capacities of 1658 and 1130 mA h g^−1^ and an outstanding cyclability of 1600 mA h g^−1^ after 500 cycles at 1 A g^−1^ (Figure 5h,i).^60^ Furthermore, Cherian et al. prepared α‐Fe_2_O_3_ NRs with an average diameter of 150 nm via an electrospinning and annealing approach using PVP/ferric acetyl acetonate (Fe(acac)_3_) precursors.^57^ The materials displayed a charge capacity of 1109 and 1095 mA h g^−1^ during the 1st and 2nd cycles_,_ respectively. In addition, the reversible capacity decreased at high rates and achieved a value of 1090 mA h g^−1^ after 70 cycles (Figure 5j,k).

3D pure Fe_2_O_3_ microspheres^58^ and flower‐like structures^59^ can be prepared by the hydrothermal and thermal decomposition methods. Zhang et al. synthesized Fe_2_O_3_ microspheres of 2 µm in diameter via a hydrothermal method (Figure 5a).^58^ An initial discharge capacity of 1477 mA h g^−1^ was achieved by Fe_2_O_3_ at 100 mA g^−1^, which was higher than that of Fe_2_O_3_ NPs (1426 mA h g^−1^). Moreover, the as‐prepared materials showed a superb reversible capacity of 705 mA h g^−1^ even over 430 cycling tests while that of the Fe_2_O_3_ NPs was only 281 mA h g^−1^ for the 130th cycle. Ma et al. fabricated flower‐like α‐Fe_2_O_3_ (Figure 5f,g) in a thermal decomposition process that is shown in Figure 5b.^59^ The α‐Fe_2_O_3_ materials were prepared differently using precursor solutions with concentrations of 0.02 (F02), 0.04 (F04), 0.06 (F06), and 0.08 m (F08). It was proved that the F02 materials with a pore size of 1.16 nm delivered an initial discharge capacity of 1522 mA h g^−1^ and exhibited a retention of 88.51% over 40 cycles, which was the highest among the F02, F04, F06, and F08 materials.

#### 3.2.2 Fe_2_O_3_/Carbon Nanomaterials

In addition to graphene and CNTs, FeO*_x_* can also form a compound with carbon. Thus, Fe_2_O_3_ NP/carbon materials,^61^, ^62^ α‐Fe_2_O_3_/carbon core–shell nanorings,^63^, ^64^ and nanocrystalline α‐Fe_2_O_3_‐loaded carbon^65^ have been studied as well.

**Figure 6:**
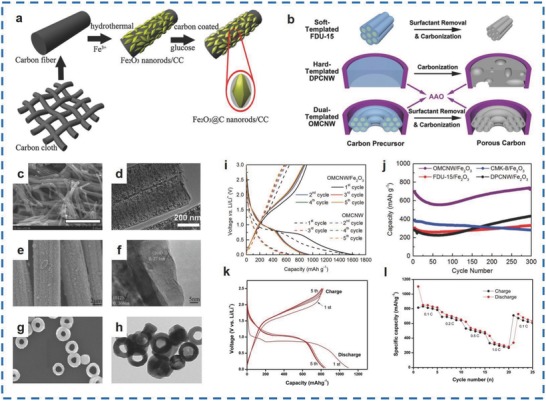
a) The synthesis procedure of Fe_2_O_3_/carbon NRs on carbon cloth. b) Schematic illustrations of different mesostructure evolutions for FDU‐15, DPCNW, and OMCNW. c) SEM image of OMCNW/Fe_2_O_3_. d) TEM image of OMCNW/Fe_2_O_3_. e) SEM image of Fe_2_O_3_/CC. f) Magnified TEM image of Fe_2_O_3_/CC. g) SEM image of α‐Fe_2_O_3_/carbon nanorings. h) TEM image of α‐Fe_2_O_3_/carbon nanorings. i) Voltage profiles of OMCNW/Fe_2_O_3_ (solid lines) and OMCNW (dashed lines) at 0.1 A g^−1^. j) Cycling performance of Fe_2_O_3_/carbon at 0.5 A g^−1^. k) Discharge/charge profiles of Fe_2_O_3_/carbon (250 µL of diamines). l) Rate performance of Fe_2_O_3_/carbon at variable C rates. (a,e,f) Reproduced with permission.^66^ Copyright 2016, Elsevier; (b,c,d,i,j) Reproduced with permission.^54^ Copyright 2016, Royal Society of Chemistry; (g,h) Reproduced with permission.^63^ Copyright 2015, Elsevier. (k,l) Reproduced with permission.^67^ Copyright 2013, Elsevier.

A hydrothermal method can be used for the synthesis of Fe_2_O_3_/carbon NRs and α‐Fe_2_O_3_/carbon nanorings.^63^, ^66^ Wang et al. fabricated Fe_2_O_3_/carbon NRs on carbon cloth via a hydrothermal approach; the first step involved loading the Fe_2_O_3_ NR arrays, and the second step involved carbon coating (**Figure** **6**a).^66^ The Fe_2_O_3_/carbon materials (Figure 6e,f) delivered initial discharge/charge capacities of 2912.6 and 2283.9 mA h g^−1^. Li et al. fabricated α‐Fe_2_O_3_/carbon nanorings with an outer diameter of 148 nm, a thickness of 50 nm, and a length of 115 nm using a hydrothermal method, and this was followed by a carbon‐coating process to form rings enwrapped with a carbon shell ≈3 nm in thickness (Figure 6g,h).^63^ This material demonstrated a specific capacity of 815 mA h g^−1^ at 1000 mA h g^−1^ over 160 cycles.

Other methods, which include the template method,^54^ modified coprecipitation method^67^ and in situ carbonization method,^68^ have also been used for the fabrication of Fe_2_O_3_/carbon materials. Hu et al. successfully synthesized ordered mesoporous carbon nanowire (OMCNW)/Fe_2_O_3_ materials (Figure 6c,d) with a template method (Figure 6b).^54^ Three kinds of ordered mesoporous carbons (OMCs), namely, FDU‐15, CMK‐8, and OMCNW, were fabricated with disordered porous carbon nanowires (DPCNWs) via the soft‐template, hard‐template, and soft‐hard dual‐template methods, respectively. The OMCNWs displayed the first lithiation/delithiation capacities of 1621 and 650 mA h g^−1^ and a lithiation capacity of 698 mA h g^−1^ during the 2nd cycle, which is higher than that of graphite (Figure 6i,j). Oh et al. fabricated Fe_2_O_3_/carbon materials using a modified coprecipitation method and found that the Fe_2_O_3_/carbon materials displayed a discharge capacity of 1094 mA h g^−1^ during the 1st cycle (Figure 6k,l).^67^ In addition, the electrode exhibited an initial charge capacity of 815 mA h g^−1^ at 0.1 C, and the capacity retention improved by 150%. Cheng et al. prepared α‐Fe_2_O_3_/carbon nanocomposites 30–35 nm in size using an in situ carbonization approach.^68^ The oleic acid‐capped α‐Fe_2_O_3_ NPs, which were applied as the precursor, were annealed at 300 °C for 2 h to prepare the α‐Fe_2_O_3_/carbon materials. As a result, the as‐prepared Fe_2_O_3_/carbon materials exhibited superb conductivity and good cycling stability. In addition, a chemical vapor deposition method was also used for the synthesis of Fe_2_O_3_ NPs/GF by Guan et al. and discharge capacities of 514.0 and 214.3 mA h g^−1^ at 5 A g^−1^ and 30 A g^−1^ were obtained, respectively.^69^ A capacity retention of 95.8% was achieved at 30 A g ^−1^ after 4000 cycles as well. In a nutshell, the carbon‐based materials have improved the conductivity of the compounds so that better performances have been achieved in LIBs.

**Figure 7:**
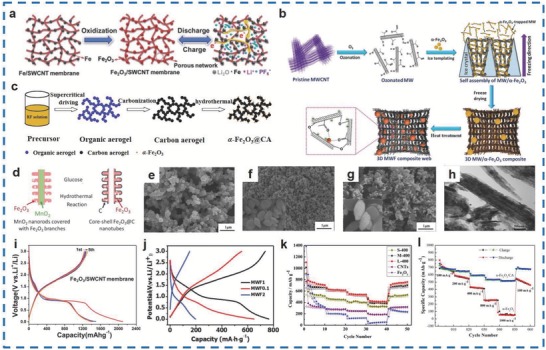
a) Schematic of the synthesis procedure and the structure of Fe_2_O_3_/SWCNT membrane. b) Synthesis procedure and structure formation mechanism of spider‐web‐like composite anode. c) Scheme of α‐Fe_2_O_3_/CA forming mechanism. d) Synthesis of branched core–shell Fe_2_O_3_/carbon nanotubes. e–g) SEM images of samples CA, bare α‐Fe_2_O_3_, and α‐Fe_2_O_3_/CA composite, respectively. The inset shows the magnification images of samples. h) TEM image of the α‐Fe_2_O_3_ nanobelts and α‐Fe_2_O_3_ nanobelts/CNTs. i) Galvanostatic charge–discharge profiles of the flexible Fe_2_O_3_/SWCNT. j) Capacity versus potential plots at 0.1 A g^−1^ for all MWF samples. k) The rate performances of S‐400, M‐400, L‐400, pure Fe_2_O_3_, and CNTs. l) Rate capability of α‐Fe_2_O_3_ and α‐Fe_2_O_3_/CA at various current rates ranged from 100 to 1000 mA g^−1^ as indicated. (a,i) Reproduced with permission.^72^ Copyright 2012, Royal Society of Chemistry; (b,j) Reproduced with permission.^73^ Copyright 2017, Wiley; (c,e–g,l) Reproduced with permission.^76^ Copyright 2017, Elsevier; (d) Reproduced with permission.^74^ Copyright 2014, Royal Society of Chemistry; (h) Reproduced with permission.^70^ Copyright 2014, Elsevier; (k) Reproduced with permission.^75^ Copyright 2013, Springer.

Fe_2_O_3_ nanobelts/CNTs,^70^ Fe_2_O_3_ NPs/CNT film materials,^71^ and other morphologies have been synthesized as well. A single‐walled Fe_2_O_3_/carbon nanotube (SWCNT) membrane was prepared by Zhou et al. with 88.0 wt% Fe_2_O_3_ NPs (5–10 nm) using floating catalyst CVD (**Figure** **7**a); the first discharge/charge capacities of the Fe_2_O_3_/SWCNT membrane were 2097 and 1243 mA h g^−1^, respectively, which were higher than those of the SWCNT membrane (Figure 7j).^72^ Furthermore, a superior reversible capacity of ≈1200 mA h g^−1^ was obtained by the Fe_2_O_3_/SWCNT membrane at 50 mA g^−1^, and the materials maintained specific capacities of 750 and 405 mA h g^−1^ even at 1000 and 2500 mA g^−1^, respectively, which were much better than that of SWCNTs (124 and 80 mA h g^−1^). Besides, γ‐Fe_2_O_3_/MWCNTs with a spider‐web‐like structure were fabricated by Bhattacharya et al. via an ozonation–templating–heating method (Figure 7b).^73^ The as‐prepared MWCNT/γ‐Fe_2_O_3_ (MWF) materials were designated as MWF0.1, MWF1, and MWF2 for the ratios of 24.05, 45.15, and 65.65 wt%, respectively. Among them, MWF1, with IR signatures of 490–800 cm^−1^, exhibited the best capacity of 766 mA h g^−1^ compared to those of MWF0.1 and MWF2 achieving 609 and 196 mA h g^−1^ at 0.1 A g^−1^, respectively (Figure 7k). It was also demonstrated that a superb discharge capacity of ≈822 mA h g^−1^ at 0.05 A g^−1^, a retention of ≈72.3% between 0.05 to 1 A g^−1^, and an outstanding cycling stability of >88% over 310 cycles (coulombic efficiency >99%) were achieved with these materials.

Apart from above studies, Gu et al. fabricated branched core–shell Fe_2_O_3_/carbon materials (Figure 7d) with different types of Fe_2_O_3_/CNT materials via a hydrothermal approach, and they were named samples I, II, and III.^74^ The three kinds of samples were wrapped with thicknesses of 0.9, 2.5, and 3.9 nm, respectively. The results show that sample I, having the thinnest carbon layer, performed best among all the as‐obtained materials and delivered high capacities of 1173 and 1014 mA h g^−1^ at 0.2 and 1 A g^−1^, respectively, over 100 cycles, while those of the other two samples were only 1025 mA h g^−1^ and 820 mA h g^−1^, 815 mA h g^−1^ and 680 mA h g^−1^. Moreover, sample I exhibited coulombic efficiencies of ≈73% and displayed a reversible capacity of 482 mA h g^−1^ at 4 A g^−1^ even over 1000 cycles. Wu et al. synthesized Fe_2_O_3_ nanobelt/CNT materials 10 nm in width by the precipitation of FeC_2_O_4_ on CNTs and a heat‐treatment process (Figure 7h,i).^70^ The Fe_2_O_3_ nanobelt/CNT materials delivered initial discharge/charge capacities of 1191.4 and 847.5 mA h g^−1^ at 100 mA g^−1^ and a reversible capacity of 865.9 mA h g^−1^ over 50 cycles and 442.1 mA h g^−1^ at 4 A g^−1^. Sun et al. fabricated Fe_2_O_3_/CNT materials containing 44.8% Fe_2_O_3_ (M‐400) through an ammonia hydrolysis and pyrolysis two‐step process.^75^ A charge capacity of 619 mA h g^−1^ was obtained by M‐400 materials over 80 cycles at 50 mA g^−1^, which achieved a superior charge capacity retention of 94.9% and even maintained 376 mA h g^−1^ at 500 mA g^−1^(Figure 7l). Overall, the electrochemical performance of the Fe_2_O_3_/CNT materials is better than that of pure Fe_2_O_3_ for applications in energy storage devices.

A hydrothermal method^76^ and sol–gel process^77^ can be used for the synthesis of Fe_2_O_3_/carbon aerogel (CA) materials. Luo et al. fabricated α‐Fe_2_O_3_/CA materials with a size of 34 nm (Figure 7e–g) through the hydrothermal process in an aqueous solution, which performed best among all the other as‐prepared materials of different sizes (Figure 7c).^76^ The α‐Fe_2_O_3_/CA materials delivered a specific capacity of 581.9 mA h g^−1^ at 100 mA g^−1^ over 50 cycling tests and a reversible capacity of 512.3 mA h g^−1^ even at 1000 mA g^−1^ (Figure 7m). Furthermore, Liu et al. fabricated Fe_2_O_3_ NPs/CA materials via a sol–gel process by soaking in a solution of Fe(NO_3_)_3_ and continuous annealing.^77^ The Fe_2_O_3_/CA‐60 materials (carbon:Fe = 48.8%:32.0%) displayed reversible capacities of 881 and 546 mA h g^−1^ at 100 and 800 mA g^−1^. Furthermore, the initial charge/discharge capacities of 916, 521 mA h g^−1^ were obtained by the as‐prepared materials, and the initial charge capacity of the as‐prepared materials was higher than those of Fe_2_O_3_ (816 mA h g^−1^) and CA (487 mA h g^−1^); however, there was no obvious change in the initial discharge capacity. It turned out that the carbon aerogel assisted the anode materials in achieving a good capacity retention, which effectively promoted their good performance in LIBs.

**Figure 8:**
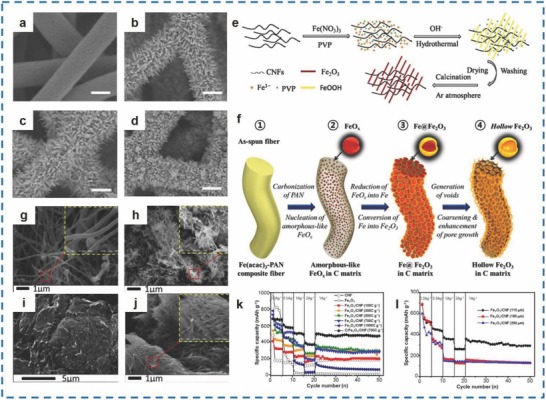
a) SEM image of bare CNFs. b–d) SEM images of the Fe_2_O_3_/CNFs composites prepared for different amounts of charge passed: 300, 500, and 1000 C g^−1^. e) Schematic preparation process of α‐Fe_2_O_3_ NRs/CNFs. f) Formation mechanism of bubble‐NR‐structured Fe_2_O_3_/carbon composite nanofiber by Kirkendall‐type diffusion. g,h) SEM images of bare CNFs and bare α‐Fe_2_O_3_ NRs. i,j) SEM images of α‐Fe_2_O_3_ NRs/CNFs. k) Rate performances of the different electrodes. l) Cycling performances of the Fe_2_O_3_/CNFs electrodes formed using CNFs with different thicknesses. (a–d,k,l) Reproduced with permission.^78^ Copyright 2015, Elsevier; (e,g–j) Reproduced with permission.^79^ Copyright 2014, Elsevier; (f) Reproduced with permission.^80^ Copyright 2015, American Chemical Society.

A precipitation method,^78^ a hydrothermal method,^79^ and an electrospinning method^80^ have also been explored to synthesize Fe_2_O_3_ NRs/carbon nanofibers (CNFs). Park et al. fabricated Fe_2_O_3_ NR/CNF materials with a diameter of 14 mm via a precipitation approach.^78^ The scanning electron microscope (SEM) images of the bare CNFs and the Fe_2_O_3_/CNF composites with a scale bar of 200 nm are shown in **Figure** **8**a–d. The Fe_2_O_3_/CNF materials delivered a specific capacity of 515.1 mA h g^−1^ at 0.5 A g^−1^, which was higher than not only the pure Fe_2_O_3_ NPs of 125.3 mA h g^−1^ but also the pure CNFs of 153.2 mA h g^−1^ (Figure 8k,l). Wu et al. synthesized α‐Fe_2_O_3_ NR/carbon materials (Figure 8g–j) with α‐Fe_2_O_3_ NRs 75 nm in diameter and 1 µm in length that were coated with CNFs via a hydrothermal method (Figure 8e).^79^ The as‐obtained materials delivered an initial discharge capacity of 1278 mA h g^−1^ at 0.2 C and maintained 960 mA h g^−1^ over 30 cycles, demonstrating better performance than pure α‐Fe_2_O_3_ NRs. Cho et al. prepared Fe_2_O_3_/carbon nanofibers with a bubble‐nanorod structure using an electrospinning method, and the hollow nanospheres exhibited a size of 17 nm and a thickness of 3 nm (Figure 8f).^80^ As a result, the pure Fe_2_O_3_ displayed initial discharge/charge capacities of 1406 and 1145 mA h g^−1^, while the as‐prepared materials were only 1385 and 975 mA h g^−1^, and in the long‐term, the capacity loss of pure Fe_2_O_3_ was greater than that of the Fe_2_O_3_/carbon nanofibers after several cycles. For example, the as‐prepared materials delivered a discharge capacity of 812 mA h g^−1^ after 300 cycles, which was higher than that of the pure Fe_2_O_3_ nanofibers (284 mA h g^−1^). In addition, the retention of each was 84 and 24%, respectively, during the 2nd cycle.

Fe_2_O_3_ NPs/graphene,^81^ Fe_2_O_3_/graphene hierarchical nanospheres,^82^ Fe_2_O_3_ nanocrystals wrapped in graphene,^83^ and core–shell nanohollow Fe_2_O_3_/graphene^84^ have also been applied in LIBs for good electrochemical performance.

**Figure 9:**
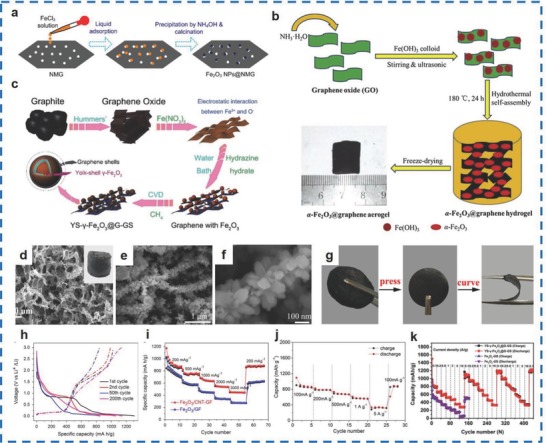
a) Schematic synthesis of Fe_2_O_3_–NMG composites. b) Schematic of the preparation of Fe_2_O_3_–GA composites. c) The schematic diagram of the synthesis procedure of YS‐γ‐Fe_2_O_3_@G‐GS. d) SEM image of the microstructure of 3D graphene/Fe_2_O_3_ prepared by annealing of 3D graphene/PB at 250 °C for 2 h. e) Low‐magnification and f) high‐magnification SEM images of Fe_2_O_3_/CNTs‐GF after 300 cycles illustrating the preservation of the core–branch structure during long cycles. g) Preparation of a flexible binder‐free 3D graphene/Fe_2_O_3_ electrode. h) Charge/discharge curves measured between 0.01 and 3.0 V at 200 mA g^−1^. i) Rate capability of Fe_2_O_3_/GF and Fe_2_O_3_/CNT/GF electrode at different current densities. j) The rate capability of P20. k) Rate cycle performance of the electrodes of YS‐γ‐Fe_2_O_3_@G‐GS and Fe_2_O_3_/GS at charge/discharge rates from 0.1 to 10 C for 460 cycles. (a) Reproduced with permission.^85^ Copyright 2014, American Chemical Society; (b) Reproduced with permission.^86^ Copyright 2016, Elsevier; (c,k) Reproduced with permission.^87^ Copyright 2017, Royal Society of Chemistry; (d,g) Reproduced with permission.^88^ Copyright 2017, American Chemical Society; (e,f, h,i) Reproduced with permission.^90^ Copyright 2014, Elsevier; (j) Reproduced with permission.^89^ Copyright 2015, Elsevier.

Different methods, such as the adsorption–precipitation method,^85^ hydrothermal method,^86^ and in situ CVD method,^87^ have been used for the fabrication of Fe_2_O_3_/graphene materials. Zhu et al. prepared Fe_2_O_3_/nanomesh graphene (NMG) materials by an adsorption and precipitation two‐step process (**Figure** **9**a).^85^ Reversible capacities of 1567 mA h g^−1^ at 150 mA g^−1^ for 50 cycles and 883 mA h g^−1^ at 1000 mA g^−1^ for 100 cycles were displayed by Fe_2_O_3_/NMG materials. Differently, Meng et al. fabricated an α‐Fe_2_O_3_ NPs/GA via a hydrothermal method (Figure 9b), which demonstrated a surface area of 212.5 m^2^ g^−1^ and a pore volume of 0.2073 cm^3^ g^−1^.^86^ Moreover, the α‐Fe_2_O_3_ NPs/GA materials showed discharge capacities of 691.9 and 187.1 mA h g^−1^ at 100 and 2000 mA g^−1^, which were higher than those of the pure Fe_2_O_3_ (358.2 and 4.4 mA h g^−1^, respectively). An in situ CVD method was used by Zhang et al. for the synthesis of yolk–shell γ‐Fe_2_O_3_ NPs encapsulated with graphene shells (YS‐g‐Fe_2_O_3_@G‐GS) (Figure 9c).^87^ The as‐prepared materials showed good cycling stability (663.7 mA h g^−1^ at 2 A g^−1^), superb rate capability (1173, 989, 827, 737, 574, 443, and 350 mA h g^−1^ at 0.1, 0.2, 0.5, 1, 2, 5, and 10 C, respectively) with a retention of ≈96.6% over 1500 cycles (Figure 9k).

In addition, Jiang et al. obtained 3D graphene/Fe_2_O_3_ materials (Figure 9d) through a combined metal‐ion‐induced spatially confined Ostwald ripening method.^88^ As shown in Figure 9g, the 3D graphene/Fe_2_O_3_ materials demonstrated flexibility; therefore, the films were compressed from ≈4 mm to ≈50 µm in thickness. The as‐fabricated materials delivered a discharge capacity of 1129 mA h g^−1^ at 0.2 A g^−1^ over 130 cycling tests and exhibited excellent cycling stability retaining 98% of the initial value over 1200 cycling tests at 5 A g^−1^. Wang et al. prepared Fe_2_O_3_ (1 nm in size) dropped with GNSs (30 nm in size) through a dielectric barrier discharge plasma assisted milling (P‐milling).^89^ It was demonstrated that the initial discharge capacities of 851 mA h g^−1^ at 100 mA g^−1^ over 5 cycles were obtained for these materials (Figure 9j). Chen et al. synthesized α‐Fe_2_O_3_ NRs dropped with a CNTs/GF, which showed good conductivity (Figure 9e,f).^90^ The materials displayed initial discharge/charge capacities of 1310 and 1028 mA h g^−1^ and, furthermore, exhibited 500 mA h g^−1^ at 3000 mA g^−1^ (Figure 9h,i). In conclusion, α‐Fe_2_O_3_/CNT/GF composites are good anode materials that can be largely applied in LIBs.

**Figure 10:**
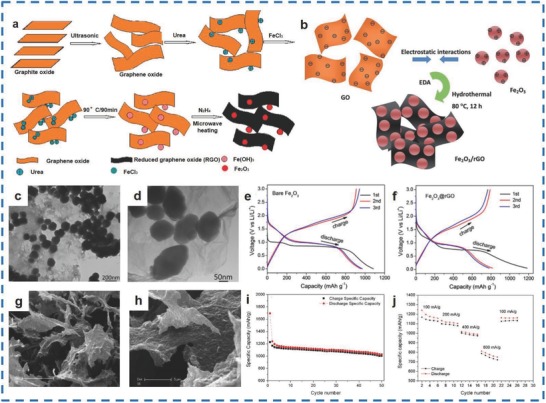
a) Scheme of Fe_2_O_3_/rGO composite forming mechanism. b) Schematic illustration of the synthesis strategy of the Fe_2_O_3_/rGO nanocomposite: electrostatic interaction induces self‐assembly that is coupled with a low‐temperature hydrothermal reduction process. c) Low‐magnification TEM images of Fe_2_O_3_–polymer. d) High‐magnification TEM images of Fe_2_O_3_/rGO composites. e,f) Discharge and charge profile for the initial 3 cycles of bare Fe_2_O_3_ and Fe_2_O_3_/rGO electrodes. g) Low magnification SEM images of the Fe_2_O_3_/rGO nanocomposite. h) High magnification SEM image of the Fe_2_O_3_/rGO nanocomposite. i) Cycling performance of Fe_2_O_3_/rGO composite at 100 mA g^−1^. j) Rate capacity of Fe_2_O_3_/rGO composite between 0.005 and 3.0 V with increasing current density. (a,i,j) Reproduced with permission.^91^ Copyright 2011, American Chemical Society; (b,g,h) Reproduced with permission.^93^ Copyright 2014, Elsevier; (c–f) Reproduced with permission.^92^ Copyright 2015, Royal Society of Chemistry.

Generally speaking, the methods for the synthesis of Fe_2_O_3_‐rGO materials mainly include the precipitation–reduction method^91^ and the hydrothermal method.^92^, ^93^ Zhu et al. fabricated Fe_2_O_3_‐rGO materials (the average diameter of the Fe_2_O_3_ particles is ≈60 nm) through a precipitation and reduction two‐step process (**Figure** **10**a).^91^ Moreover, initial discharge/charge capacities of 1693 and 1227 mA h g^−1^ were achieved for the as‐synthesized materials at 100 mA h g^−1^. In addition, the materials had a high capacity retention of 1027 mA h g^−1^ over 50 cycles, and the discharge capacity was maintained at ≈800 mA h g^−1^ at 800 mA g^−1^ (Figure 10i,j). Xiao et al. synthesized Fe_2_O_3_ NPs of 9 nm in diameter decorated on rGO sheets (Figure 10g,h) via a hydrothermal reduction process without any surfactant or chemical linker (Figure 10b).^92^ Specific capacities of 600 and 180 mA h g^−1^ were achieved for the Fe_2_O_3_/rGO materials at 0.1 and 10 A g^−1^, respectively. Similarly, Chen et al. fabricated Fe_2_O_3_ NPs (with an average size of ≈180 nm) dropped on rGO sheets (Figure 10c,d) using a hydrothermal approach.^93^ The initial discharge capacity of the Fe_2_O_3_/rGO material was 1186 mA h g^−1^, which was higher than pure Fe_2_O_3_ with a specific discharge capacity of 1085 mA h g^−1^. In addition, the materials delivered a reversible capacity of 930 mA h g^−1^, while pure Fe_2_O_3_ exhibited a reversible capacity of only 910 mA h g^−1^. This result is due to the rGO nanosheets promoting electron transport, improving the conductivity of the materials.

Based on above binary composites, ternary composites of carbon‐wrapped Fe_2_O_3_ particles deposited on graphene nanosheets (GNSs),^94^ α‐Fe_2_O_3_/carbon/graphene microspheres,^95^ and Fe_2_O_3_/graphene/CNT films^96^ have been explored. Zhang et al. proposed a spray drying–carbonization–oxidation method to synthesize α‐Fe_2_O_3_/graphene/carbon microspheres with α‐Fe_2_O_3_ NPs and graphitic carbon shells 30–50 nm in size and 5–10 nm in thickness.^95^ The initial discharge/charge capacities of 1363 and 898 mA h g^−1^ were achieved at 400 mA g^−1^. In addition, the as‐prepared materials exhibited a coulombic efficiency of 66% and even maintained 841 mA h g^−1^ over 100 cycles. Wang et al. prepared Fe_2_O_3_/carbon/GN materials, which were wrapped with a carbon layer with a thickness of 5 nm on Fe_2_O_3_ particles 400 nm in size via a hydrothermal and glucose impregnation–pyrolysis two‐step process.^94^ The as‐obtained materials exhibited the initial discharge/charge capacities of 1540 and 1100 mA h g^−1^ at 200 mA g^−1^, which were higher than those of pure Fe_2_O_3_ (1288 and 706 mA h g^−1^) and Fe_2_O_3_/GNs (1475 and 936 mA h g^−1^) and the coulombic efficiency of the materials was up 71%. Flexible Fe_2_O_3_/graphene/CNTs films with the hierarchical structure were fabricated by Wang et al. with pure Fe_2_O_3_, which was 750 nm in diameter through filtration and reduction two processes. As a result, the materials showed a reversible capacity of 716 mA h g^−1^ at 50 mA g^−1^ at the 120th cycle.^96^

#### 3.2.3 Fe_2_O_3_/Metal‐Based Nanomaterials

Apart from the Fe_2_O_3_/carbon materials, Fe_2_O_3_/metal‐based materials such as γ‐Fe_2_O_3_/Ag NW nanocables^97^ and α‐Fe_2_O_3_/Li/Fe materials^98^ have been studied. Geng et al. fabricated γ‐Fe_2_O_3_/Ag NW materials 90 nm in diameter through a mild oxidation method.^97^ As a result, the materials exhibited a reversible capacity of 890 mA h g^−1^ over 60 cycling tests at 0.1 C and maintained 550 mA h g^−1^ even at 2.0 C. Differently, α‐Fe_2_O_3_ (with a crystallite size of 50 nm)/Li/Fe (with a crystallite size of 29 nm) materials were synthesized by Wang et al. through a gel polymer method.^98^ The capacities of 1300 and 1400 mA h g^−1^ were obtained by α‐Fe_2_O_3_ and Li/Fe oxides, respectively, for which the initial capacitance loss resulted in a retention of only 21.8%.

**Figure 11:**
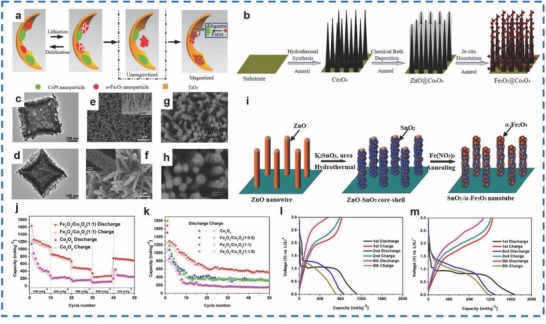
a) The proposed model of the internal magnetic field for reusing pulverized α‐Fe_2_O_3_ during lithiation/delithiation process. b) A schematic illustration of the formation of hierarchical Fe_2_O_3_/Co_3_O_4_ nanowire array. c) TEM image of Fe_2_O_3_/Co_3_O_4_ hollow microcubes with molar ratio for Fe/Co = 1:0.5. d) TEM image of Fe_2_O_3_/Co_3_O_4_ hollow microcubes with molar ratio for Fe/Co = 1:1. e,f) Hierarchical Fe_2_O_3_/Co_3_O_4_ nanowire array. g,h) SnO_2_/α‐Fe_2_O_3_ composite nanotube array. i) Schematic illustration of the fabrication process of the composite nanotube array electrode. j) Rate capability of the Fe_2_O_3_/Co_3_O_4_ and pure Co_3_O_4_ electrodes cycled at different current densities from 100 to 800 mA g^−1^ in the voltage range 3.00–0.01 V versus Li^+^/Li for 50 cycles. k) Discharge and charge capacity versus cycle numbers curves of the Fe_2_O_3_/Co_3_O_4_ and pure Co_3_O_4_ electrodes cycled at a current density of 100 mA g^−1^ in the voltage range 3.00–0.01 V versus Li^+^/Li for 50 cycles. l,m) Discharge–charge profiles of electrode at a current density of 100 mA g^−1^ in the voltage range 3.00–0.01 V versus Li^+^/Li: pure Co_3_O_4_ NPs, the Fe_2_O_3_/Co_3_O_4_ (1:1) hollow microcubes, respectively. (a) Reproduced with permission.^103^ (b,e,f) Reproduced with permission.^101^ Copyright 2013, Elsevier; Copyright 2017, Royal Society of Chemistry; (c,d,j–m) Reproduced with permission.^100^ Copyright 2014, American Chemical Society; (g–i) Reproduced with permission.^104^ Copyright 2012, Royal Society of Chemistry.

Generally, Fe_2_O_3_/Co_3_O_4_ materials can be synthesized by hydrothermal and hydrolysis methods^99^ or annealing and chemical reactions.^100^ Xiong et al. synthesized Fe_2_O_3_/Co_3_O_4_ NW arrays with a nanocrystallite size of 10–20 nm and a pore size of 4–6 nm (**Figure** **11**e,f) via hydrothermal and hydrolysis methods.^101^ Figure 11b shows the growth of Co_3_O_4_ nanowire arrays on a Ni substrate through a hydrothermal approach. The as‐prepared materials displayed a cyclability of 1005.1 mA h g^−1^ over 50 cycling tests at 200 mA g^−1^ and a rate capacity of 788.9 mA h g^−1^ at 5000 mA g^−1^. α‐Fe_2_O_3_/Co_3_O_4_ branched NWs with 50–100 nm in diameter and length were prepared by Wu et al. via a hydrothermal approach, by which a first discharge capacity of ≈1534 mA h g^−1^ was achieved at 100 mA g^−1^, while those of the Co_3_O_4_ NWs and α‐Fe_2_O_3_ were only 1188 and 155 mA h g^−1^, respectively.^99^ Furthermore, the as‐prepared materials maintained a reversible capacity of 980 mA h g^−1^ from the 2nd cycle to 60th cycle, which was higher than those of the Co_3_O_4_ NWs (311 mA h g^−1^) and α‐Fe_2_O_3_ NWs (75 mA h g^−1^), and the capacitance retention was as high as 66% of the initial discharge capacity. Wang et al. fabricated Fe_2_O_3_ NRs 10 nm in diameter on SnO_2_ nanosheets via a hydrothermal growth method.^102^ The as‐prepared materials exhibited an initial discharge capacity of 1632 mA h g^−1^ at 400 mA g^−1^ and maintained a capacity of 325 mA h g^−1^ over 50 cycles. Li et al. fabricated Fe_2_O_3_/Co_3_O_4_ double‐shelled hierarchical microcubes with an 800 nm average size by annealing Fe_4_[Fe(CN)_6_]_3_/Co(OH)_2_ microcubes and then reacting Co^2+^ (from Co(AC)_2_) with OH^−^ (from the reaction of ammonium hydroxide and water) (Figure 11c,d); this material delivered discharge/charge capacities of 1678 and 1249 mA h g^−1^, which were higher than those of bare Fe_2_O_3_ (1108 and 806 mA h g^−1^).^100^ In addition, 456 mA h g^−1^ at 400 mA g^−1^ and 272 mA h g^−1^ at 800 mA g^−1^ were achieved for Fe_2_O_3_/Co_3_O_4_ materials as well (Figure 11j–m).

In addition to the abovementioned methods, a template method has been used to prepare other Fe_2_O_3_/metal‐based materials. The 3D ordered macroporous TiO_2_/CoPt/α‐Fe_2_O_3_ materials were fabricated by Tang et al. using a sol–gel method, which was templated by poly (methyl methacrylate) microspheres (Figure 11a).^103^ The initial discharge/charge capacities of 1563 and 650 mA h g^−1^ were achieved for the as‐synthesized materials at 50 mA g^−1^. Zeng et al. prepared an α‐Fe_2_O_3_/SnO_2_ nanotube array 200–400 nm in diameter (Figure 11g,h) using a template method with a ZnO nanowire array template (Figure 11i),^104^ and the synthesized array displayed an excellent discharge capacity of 0.727 mA h cm^−2^ over 50 cycles at 0.1 mA cm^−2^ during the 1st cycle. In addition, Wang et al. and Zhou et al. also synthesized α‐Fe_2_O_3_/SnO_2_ materials. However, Zhou et al. synthesized α‐Fe_2_O_3_/SnO_2_ materials by growing SnO_2_ nanorods 150 and 20 nm in length and diameter on Fe_2_O_3_ nanoflakes 10 nm in thickness to achieve a tree‐like branched structure.^105^ As a result, an areal capacity of 0.43 mA h cm^−2^ was obtained by the as‐prepared materials after 150 cycles, which was higher than that of pure Fe_2_O_3_ nanoflakes (0.25 mA h cm^−2^). Hence, Fe_2_O_3_/SnO_2_ materials have been demonstrated as a promising anode material for LIBs.

Fe_2_O_3_/SnO_2_/carbon materials can be fabricated through a hydrothermal process^106^ or in situ polymerization.^107^ Du et al. fabricated porous γ‐Fe_2_O_3_/SnO_2_/carbon NRs through a hydrothermal method, the precursors of which were FeOOH NRs with 20–40 nm in diameter and 200 nm in length and SnO_2_ with 5–10 nm in thickness.^106^ It was demonstrated that initial discharge/charge capacities of 1211.1 and 893.7 mA h g^−1^ were achieved for the as‐prepared materials at 200 mA g^−1^, and the coulombic efficiency was ≈73% during the 1st cycle. Furthermore, the γ‐Fe_2_O_3_/SnO_2_/carbon NRs delivered a reversible capacity of 879 mA h g^−1^ over 60 cycles and a capacitance retention of ≈88%, demonstrating better electrochemical performance compared with SnO_2_ and Fe_2_O_3_/SnO_2_. Additionally, in situ polymerization was applied for the synthesis of SnO_2_/Fe_2_O_3_/carbon materials by Guo et al.^107^ As a result, the as‐synthesized materials delivered the first discharge/charge capacities of 2506 and 1606 mA h g^−1^ at 200 mA g^−1^, which were higher than those of the Fe_2_O_3_/SnO_2_ NPs (1175 and 621 mA h g^−1^). Moreover, the Fe_2_O_3_/SnO_2_/carbon materials exhibited an initial coulombic efficiency of 64%, while that of Fe_2_O_3_/SnO_2_ NPs was only 53% due to the carbon layer.

Fe_2_O_3_/SnO_2_/graphene materials can be prepared by filtration–thermal reduction method^108^ and physical blending method.^109^ Liu et al. decorated Fe_2_O_3_/SnO_2_ NPs on graphene films with a precursor 40–70 nm in diameter and 200–400 nm in length and SnO_2_ NPs of 5 nm in size via filtration–thermal reduction.^108^ The as‐prepared materials delivered initial discharge/charge capacities of 2063 and 1255 mA h g^−1^ at 100 mA g^−1^, which were the highest among those of Fe_2_O_3_ (1007 mA h g^−1^), SnO_2_ (782 mA h g^−1^), and graphene (372 mA h g^−1^), and maintained a reversible discharge capacity of 1015 mA h g^−1^ over 200 cycles. Lin and Wang prepared Fe_2_O_3_/SnO_2_/GN films using a physical blending approach, and the films exhibited an initial discharge capacity of 946 mA h g^−1^ at 100 mA g^−1^ and retained a value of 538 mA h g^−1^ over 90 cycles.^109^ In addition, Fe_2_O_3_/SnO_2_/rGO materials were fabricated by Xia et al. via a precipitation approach with Fe_2_O_3_ NPs with ≈20 nm in size, which exhibited the first discharge/charge capacities of 1179 and 746 mA h g^−1^ at 400 mA g^−1^.^110^ Besides, the as‐synthesized materials exhibited a discharge capacity of ≈700 mA h g^−1^ after 100 cycling tests.

### 3.3 SIBs

Pure Fe_2_O_3_, Fe_2_O_3_/graphene Fe_2_O_3_/rGO materials have also been synthesized by different methods for good performance in SIBs.

γ‐Fe_2_O_3_ films were fabricated by Sun et al. via radio frequency magnetron sputtering by decorating γ‐Fe_2_O_3_ films on Cu sheet substrates.^111^ It was demonstrated that the initial discharge/charge capacities of 570 and 510 mA h g^−1^ were achieved at 500 mA g^−1^ for the γ‐Fe_2_O_3_ films annealed at 600 °C, which maintained nearly 100% of the initial reversible capacity over 100 charge/discharge cycles. Apart from that, Li et al. prepared the Fe_2_O_3_/GNSs via a chemical reaction–oxidation method by growing Fe_2_O_3_ NPs (5 nm in diameter) on graphene nanosheets.^23^ As a result, the as‐prepared materials exhibited the first discharge/reversible capacities of 542 and 440 mA h g^−1^ by Fe_2_O_3_/GNS at 100 mA g^−1^ and showed 81.2% of the first coulombic efficiency at 250 mA h g^−1^. Furthermore, a specific capacity of 440 mA h g^−1^ was achieved at 100 mA g^−1^, which was higher than that of crystalline Fe_2_O_3_ with a specific capacity of 284 mA h g^−1^. Furthermore, the as‐synthesized materials delivered a specific capacity of 219 mA h g^−1^ even at 2 A g^−1^. Fe_2_O_3_ nanocrystals with a particle size of ≈2 nm were decorated on GNSs by Jian et al. using a nanocasting approach.^112^ It was demonstrated that a capacity of ≈400 mA h g^−1^ was achieved and maintained by Fe_2_O_3_/GNS at 100 mA g^−1^ after 200 cycling tests and the materials delivered a capacity of 190 mA h g^−1^ at 1000 mA g^−1^, which was ≈45% of that at 100 mA g^−1^.

The microwave methodology has also been applied in the synthesis of Fe_2_O_3_/rGO materials.^22^, ^113^ Zhang et al. fabricated α‐Fe_2_O_3_/rGO materials with α‐Fe_2_O_3_ NPs (50 nm in diameter) and GNSs via a microwave hydrothermal method to deliver a discharge capacity of ≈310 mA h g^−1^ over 150 cycles at 100 mA g^−1^.^22^ In addition, a microwave‐assisted reduction method was applied by Liu and co‐workers to prepare Fe_2_O_3_/rGO materials.^113^ It was demonstrated that the Fe_2_O_3_/30 wt% rGO materials exhibited a reversible capacity of 389.3 mA h g^−1^, which was higher than that of bare Fe_2_O_3_/30 wt% rGO of 287.3 mA h g^−1^ (59% of the initial coulombic efficiency). As a result, a superior reversible capacity of 289 mA h g^−1^ was obtained by the Fe_2_O_3_ with 30 wt% rGO at 50 mA g^−1^ over 50 cycles. In summary, the pure Fe_2_O_3_ and Fe_2_O_3_/carbon materials were also superb anode materials for applications as energy storage materials in SIBs.

### 3.4 Others

Fe_2_O_3_/nonmetal‐based materials, which include B (Boron)‐containing Fe_2_O_3_ nanocomposites^114^ and Fe_2_O_3_/Se composite nanorods,^115^ have been explored.

**Figure 12:**
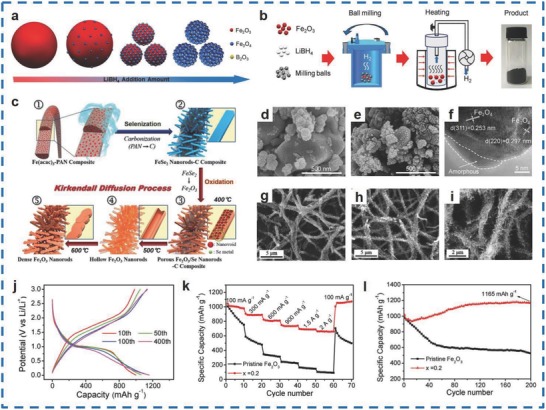
a) Morphology B‐containing Fe_2_O_3_ nanocomposites. b) Schematic of the preparation process of B‐containing Fe_2_O_3_ nanocomposites. c) Formation mechanism of the 1D nanostructure comprising porous Fe_2_O_3_/Se composite and subsequent hollow Fe_2_O_3_ NRs by nanoscale Kirkendall diffusion. d) SEM image of pristine Fe_2_O_3_. e) SEM image of B‐containing Fe_2_O_3_ nanocomposite sample (*x* = 0.2). f) High‐resolution TEM image of the B‐containing sample (*x* = 0.2). g–i) Morphologies of the 1D nanostructures comprising nanorods obtained after selenization at 400 °C and subsequent oxidation at 400, 500, and 600 °C. j) Charge/discharge curves at different cycles for the B‐containing (*x* = 0.2) sample at 100 mA g^−1^. k) Specifc capacity of pristine Fe_2_O_3_ and B‐containing (*x* = 0.2) samples obtained for cycles at different current densities. l) Cycling performance curves of pristine Fe_2_O_3_ and B‐containing samples prepared from Fe_2_O_3_‐*x*NaBH_4_. (a,b,d,e,j–l) Reproduced with permission.^114^ Copyright 2017, Wiley; (c,g,i) Reproduced with permission.^115^ Copyright 2017, Royal Society of Chemistry.

Generally speaking, ball milling–heating^114^ and oxidation methods^115^ are used for the fabrication of the two different types of materials. Cao et al. synthesized B‐containing Fe_2_O_3_ materials using the ball milling–heating method with the Fe_2_O_3‐_ *_x_*LiBH_4_ composites (**Figure** **12**a,b).^114^ As shown in Figure 12d–f, pure Fe_2_O_3_ exhibited smooth surfaces, while the as‐prepared materials, which were designed as B‐containing nanocomposites for *x* = 0.1, 0.2, 0.3, and 0.4 (with coulombic efficiencies of 74.6%, 75.9%, 77.2%, and 78.1%, respectively), had NPs with a size of ≈10 nm decorated on their surfaces. The discharge/charge potential profiles of the B‐containing nanocomposites for *x* = 0.2 remained constant from 10 to 500 cycles, and the materials displayed a specific capacity of 660 mA h g^−1^ at 2 C, which was higher than that of pure Fe_2_O_3_ with 210 mA h g^−1^ (Figure 12j,k). In addition, the Fe_2_O_3_‐0.2NaBH_4_ materials maintained a capacity of 1165 mA h g^−1^ over 200 cycles at 100 mA g^−1^ (Figure 12l). Furthermore, Cho et al. obtained Fe_2_O_3_/Se nanorods via an oxidation method.^115^ The as‐prepared materials showed different crystallite sizes of 16, 28, and 39 nm when post‐treated at 400, 500, and 600 °C, and the samples were designated as Sel.400‐Oxi.400, Sel.400‐Oxi.500, and Sel.400‐Oxi.600, respectively (Figure 12g–i). In addition, it was demonstrated that Sel.400‐Oxi.400 showed the best electrochemical performance among the three samples and delivered an initial discharge capacity of 1458 mA h g^−1^, which was higher than the others of 1303 and 1193 mA h g^−1^ at 1 A g^−1^. In conclusion, the as‐prepared materials are good candidates for LIBs.

Generally, pure α‐Fe_2_O_3_ and Fe_2_O_3_/rGO materials can be fabricated by a hydrothermal approach in LSBs. Rao et al. synthesized α‐Fe_2_O_3_ particles with 20–30 nm in size through a microwave‐assisted hydrothermal method.^116^ It was demonstrated that initial discharge/charge capacities of 1364 and 1031 mA h g^−1^ were obtained by the pure α‐Fe_2_O_3_, and it exhibited a reversible capacity of 1000 mA h g^−1^ during the 2nd cycle, showing a capacitance retention of 75.5%. Analogously, α‐Fe_2_O_3_ nanorods with a length of 400 nm and a diameter of ≈80 nm anchored on rGO nanosheets (α‐Fe_2_O_3_/rGO NRAs) were synthesized by Kong et al. via a hydrothermal method, as well.^117^ As a consequence, the as‐prepared materials delivered the first discharge/charge capacities of 832.0 and 402.4 mA h g^−1^ at 200 mA g^−1^, showing a coulombic efficiency of 48.2%. In addition, the α‐Fe_2_O_3_/rGO NRAs showed a specific capacity of about 420 mA h g^−1^ at 0.1 C, with ≈92 mA h g^−1^ even at 1.6 C.

Similarly, bare α‐Fe_2_O_3_ microparticles^118^ and Fe_2_O_3_ NPs/carbon^119^ have been studied for Fe–air batteries. Hang et al. fabricated Fe_2_O_3_ with rhombohedra structure with α‐Fe_2_O_3_ (1–10 µm in size) via a modified polyol method, and this material exhibited an initial discharge capacity of ≈320 mA h g^−1^.^118^ Besides, chemical methods were used by Hang and Thang to synthesize Fe_2_O_3_/carbon materials by decorating Fe_2_O_3_ NPs on different types of carbon and it was demonstrated that when employed with tubular CNFs, AB, and graphite, the materials exhibited superb electrochemical performance.^119^

In conclusion, a brief comparison of the synthesis methods and electrochemical performances of the Fe_2_O_3_‐based materials for rechargeable batteries is shown in **Table** **2**. It is obvious that, after improving the synthesis approaches and preparing different kinds of Fe_2_O_3_‐based materials, the capacities have increased compared to pure Fe_2_O_3_.

**Table 2:** Fe_2_O_3_‐based materials as anodes for rechargeable batteries

Materials	Methods	CDa) [C or mA g^−1^] DC/CCb) [mA h g^−1^]	Capacity [mA h g^−1^]/Cycles
**LIBs**			
Fe_2_O_3_ 120	Hydrothermal, annealing	40,–,–	900.2,35
3D network structured Fe_2_O_3_ 121	Chemical corrosion, thermal oxidation	C/0.2,895.4,680.6	926.1,400
Fe_2_O_3_ NRs^122^	Solution‐phase growth, chemical etching	C/1,1238,904	950,100
Fe_2_O_3_@carbon^68^	Surfactant carbonization	C/0.2,1300,–	688,50
Fe_2_O_3_@carbon^123^	Molten salt process, dispersion in toluene	C/2,1858,–	2112,100
Fe_2_O_3_@carbon NFs^124^	Electrospinning	C/0.2,–,–	820,100
Fe_2_O_3_@MWCNTs^125^	Hydrothermal, annealing	100,1256,700	430,100
Fe_2_O_3_ NRs@CNF^126^	Hydrothermal, annealing	201,1278,896	758,50
Fe_2_O_3_ particles@graphene^127^	Hydrothermal, annealing	50,1561,1206	1069,50
Fe_2_O_3_ particles@rGO^128^	Hydrothermal, annealing	100,1578,1095	950,70
Fe_2_O_3_ NPs@rGO^129^	Microwave heating	1000,979,–	650,50
Fe_2_O_3_@carbon@graphene microspheres^95^	Drying, carbonization, oxidation	400,1363,–	841,100
Fe_2_O_3_@Fe NPs@graphene^130^	Hydrothermal, in situ thermal reduction	100,1109.8,2147.5	959.3,90
Fe_2_O_3_@Co_3_O_4_ nanowires^101^	Hydrothermal, hydrolysis	200,1586.9,–	1005.1,50
Fe_2_O_3_@SnO_2_ porous nanocubes^131^	Solvothermal, annealing	200,–,–	567.5,50
Fe_2_O_3_@SnO_2_@carbon^107^	In situ polymerization in sol, carbonization	400,2506,1606	1000,380
Fe_2_O_3_@SnO_2_@graphene films^109^	Mixing, filtering, freeze‐drying, annealing	100,946,–	538,90
Fe_2_O_3_ NPs@SnO_2_@rGO^110^	Precipitation, reduction	400,1179,746	700,100
Fe_2_O_3_ NRs@N‐doped graphene^132^	Hydrothermal, annealing	2000,–,–	508,200
**SIBs**			
Fe_2_O_3_ films^133^	Cu template, magnetron sputtering	500,650,425	450,100
Fe_2_O_3_ nanocrystals@graphene nanosheets^112^	Nanocasting technique	100,1103,535	400,200
Fe_2_O_3_@graphene nanosheets^23^	Chemical reaction–oxidation	542,100, –	110,500
α‐Fe_2_O_3_@rGO^112^	Hydrothermal, annealing	–,–,–	310,150
Fe_2_O_3_@rGO^113^	Microwave‐assisted reduction	–,–,–	289,50
**LSBs**			
Fe_2_O_3_ 24	Hydrothermal, annealing	C/0.1,1364,1031	799,30
Fe_2_O_3_ 134	Pyrolysis	–,–,–	–,–
α‐Fe_2_O_3_ nanorod@rGO^117^	Seed‐assisted hydrothermal, annealing	200,1837.6,1238.2	1200,500

^a)^ CD: Current density (C or mA g^−1^)

^b)^ DC/CC: Initial discharge/charge capacity (mA h g^−1^).

## 4 Fe_3_O_4_‐Based Nanomaterials

### 4.1 Supercapacitors

Fe_3_O_4_‐based materials, which include pure Fe_3_O_4_, Fe_3_O_4_/carbon materials, and Fe_3_O_4_/metal‐based materials (metal or metal oxide)/carbon materials, have been explored in SCs.^32^, ^135^, ^136^

#### 4.1.1 Pure Fe_3_O_4_

Pure Fe_3_O_4_ NPs and Fe_3_O_4_ thin films can be synthesized through different strategies, which include hydrolysis and the hydrothermal method.^137^, ^138^ Wang et al. prepared Fe_3_O_4_ NPs with 5–10 nm in diameter by using FeCl_3_ and the organic solvent ethanolamine.^137^ It was demonstrated that a superb capacitance of 207.7 F g^−1^ at 0.4 A g^−1^, a good rate performance (90.4 F g^−1^ at 10 A g^−1^), and a capacity retention of 100% over 2000 cycling tests were obtained for Fe_3_O_4_ NPs. Similarly, a hydrothermal method was also adopted by Chen et al. for the synthesis of the Fe_3_O_4_ films with particle size of 300 nm–1 µm.^138^ As a result, the Fe_3_O_4_ films delivered a superb capacitance of 118.2 F g^−1^ at 6 mA g^−1^ and showed a capacitance retention of 88.75% over 500 cycling tests.

#### 4.1.2 Fe_3_O_4_/Carbon Nanomaterials

Generally, strategies for synthesizing Fe_3_O_4_/carbon materials mainly include the reduction method,^139^ chemical coprecipitation method,^140^ solvothermal/hydrothermal–calcination/sintering method,^141^, ^142^ electrospinning technique,^143^ solvent‐thermal method, and the microwave method.^144^

**Figure 13:**
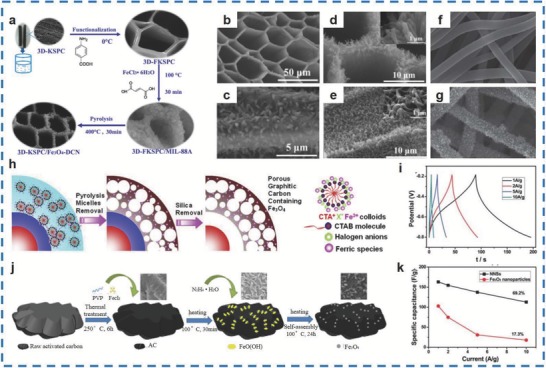
a) Schematic illustration of the formation process of 3DKSPC/Fe_3_O_4_‐DCN nanocomposites. b,c) SEM images of 3D‐KSPC/MIL‐88A of top view and side view. d,e) SEM images of 3D‐KSPC/Fe_3_O_4_‐DCN obtained at 400 °C for 30 min of top view and side view. f) SEM image of the pure CNFs. g) Low‐magnification SEM image of the Fe_3_O_4_/CNFs nanocomposites. h) Formation mechanism of double‐shelled Fe_3_O_4_/carbon/carbon hollow spheres with porous structure caused by the local melting of the micelle colloids (CTA^+^X^−1^Fe^3+^). i) Charge/discharge curves of NNSs at different current densities. j) Schematic illustration of synthesis of Fe_3_O_4_ on oxidized activated carbon. k) Comparison of the specific capacitance of NNSs and Fe_3_O_4_ NPs at various current densities. (a–e) Reproduced with permission.^148^ Copyright 2016, American Chemical Society; (f,g) Reproduced with permission.^143^ Copyright 2011, Royal Society of Chemistry; (h) Reproduced with permission.^146^ Copyright 2016, Elsevier; (i–k) Reproduced with permission.^147^ Copyright 2013, Royal Society of Chemistry; (l) Reproduced with permission.^139^ Copyright 2014, Elsevier.

The Fe_3_O_4_/carbon core–shell microspheres,^145^ Fe_3_O_4_‐doped double‐shelled hollow carbon spheres,^146^ and Fe_3_O_4_ NPs/carbon^144^ have been explored. Liu et al. fabricated Fe_3_O_4_/carbon nanosheets (NNSs) with a pore size of >100 nm and a specific surface area of 229 m^2^ g^−1^.^147^ The as‐synthesized materials displayed a superb capacitance of 163.4 F g^−1^ at 1 A g^−1^, maintained 113 F g^−1^ even at 10 A g^−1^ and demonstrated a retention of 69.2% (**Figure** **13**i,k). In addition, Wang and co‐workers synthesized Fe_3_O_4_‐doped porous carbon nanorods/3D kenaf stem‐derived macroporous carbon (Fe_3_O_4_‐DCN/3D‐KSPC) (Figure 13d,e) via a pyrolyzation‐annealing method (Figure 13a).^148^ Figure 13b,c shows the top and side views of 3D‐KSPC with the structure of iron fumarate metal organic frameworks (MIL‐88A). The as‐synthesized materials delivered a superb capacitance of 285.4 F g^−1^ at 1 A g^−1^ and retained a specific capacitance of 220.5 F g^−1^ at 2 A g^−1^ even over 5000 cycles. Additionally, Li et al. fabricated Fe_3_O_4_/carbon/carbon hollow spheres, as shown in Figure 13h, with a hierarchical pore network structure by decorating Fe_3_O_4_ species on a carbon coating.^146^ As a result, the as‐prepared materials displayed a high capacitance of 1153 F g^−1^ at 2 A g^−1^ and a good rate capability of 514 F g^−1^ at 100 A g^−1^. Oh et al. synthesized the oxidized activated carbon/Fe_3_O_4_ (AC/Fe_3_O_4_) through a reduction method, depositing Fe_3_O_4_ NPs with a diameter of 30 nm in poly(vinylpyrrolidone) on AC (Figure 13j).^139^ A specific capacitance of 202.6 F g^−1^ was achieved by the AC/Fe_3_O_4_ materials, which was higher than pure Fe_3_O_4_ (99.4 F g^−1^) at 10 mV s^−1^, and it maintained 94% of the initial value even over 5000 cycles. An electrospinning technique and a solvent‐thermal method were used by Mu and co‐workers for the synthesis of Fe_3_O_4_ nanosheets on CNFs with 400–500 nm in diameter (Figure 13f,g).^143^ As a result, the as‐fabricated Fe_3_O_4_/CNFs delivered a specific capacitance of 135 F g^−1^ while that of bare Fe_3_O_4_ was only 83 F g^−1^, and the materials showed a capacity retention of 91% over 1000 cycles. In addition, Yun et al. prepared the γ‐Fe_3_O_4_ nanobox hybrids (γ‐Fe NBhs) via a phase controlled solution method and the γ‐Fe_3_O_4_ is reduced from the α‐Fe_2_O_3_.^149^ It was demonstrated that the specifc capacities of the γ‐Fe NBhs decreased from 497.7 to 210.3 mA h g^−1^ while those of hierarchically structured rGO/α‐Fe_2_O_3_ (rGO/α‐Fe) diminished from 662.6 at 50 mA g^−1^ to 83.6 mA h g^−1^ at 1000 mA g^−1^. By contrast, the capacity retention of γ‐Fe NBhs was 42.3%, which was higher than that of rGO/α‐Fe (12.6%) over 60 cycles at 100 mA g^−1^. It is the phase transition that contributes to the differences between the two as‐prepared materials on the morphology, structure, and electrochemical performances. So, the influences of phase transition should be paid more attention to.

The graphene/Fe_3_O_4_ NPs^150^, ^151^ or NRs^152^ have also been explored through hydrothermal method,^150^ a vacuum filtration–drying–peeling off method,^151^ an ultrasonication method,^152^ and a coprecipitation method.^153^ Liu et al. prepared Fe_3_O_4_/graphene sheets (GSs) by the vacuum filtration–drying–peeling off method, which grew Fe_3_O_4_ NPs of 5 nm on GSs.^151^ It was demonstrated that the ideal Fe_3_O_4_/GSs (64.8%) with a specific surface area of 310 m^2^ g^−1^ delivered superb capacitances of 368 F g^−1^ at 1 A g^−1^ and 225 F g^−1^ at 5 A g^−1^. Lu et al. fabricated Fe_3_O_4_/rGO materials with Fe_3_O_4_ NPs (20 nm in diameter) through a coprecipitation method, the specific surface area of which was 147 m^2^ g^−1^.^153^ Not only a superb energy density of 43.2 W h kg^−1^ at 272.8 W kg^−1^ but also a good power density of 2183.5 W kg^−1^ at 27.9 W h kg^−1^ were observed. Das et al. synthesized Fe_3_O_4_ NRs 150 nm in size decorated on rGO materials (186 m^2^ g^−1^ in specific surface area) with rod structures using an ultrasonication method, which displayed a specific capacity of 315 C g^−1^ at 5 A g^−1^ and exhibited an outstanding cyclability retention of 95% over 2000 cycles.^152^ Fe_3_O_4_ NPs with 5 nm in diameter were anchored on rGO by Li et al. via a hydrothermal method and displayed an excellent capacitance of 241 F g^−1^ at 1 A g^−1^.^150^ In addition, a cyclability of 79.2% was also observed over 1000 cycling tests at 10 A g^−1^. Hence, the Fe_3_O_4_/graphene materials demonstrate a promising future in applications for SCs.

#### 4.1.3 Fe_3_O_4_/Carbon/Metal‐Based Nanomaterials

Fe_3_O_4_/metal‐based materials/carbon including Fe_3_O_4_/Fe/CNTs^154^ and Fe_3_O_4_/MnO_2_/carbon materials^155^ have been synthesized via a chemical synthesis method^154^ and electrodeposition method^155^ for SCs, respectively.

Fe_3_O_4_/Fe/CNTs with 20–30 nm outer diameters were prepared by Sun et al. via a chemical synthesis method, and they displayed specific capacitances of 85.29, 70.58, 58.8, 50, and 40.2 F g^−1^ at 0.5, 1, 2, 5, and 10 A g^−1^, respectively.^154^ In addition, excellent capacitances of 1065 F g^−1^ at 1 A g^−1^ and 595.2 F g^−1^ at 5 A g^−1^ and a retention of 82.1% at 1 A g^−1^ over 1000 cycles were achieved by the as‐prepared materials, and the retention is higher than that of Fe_3_O_4_ nanosheets of 62.3%. Apart from the Fe_3_O_4_/Fe/CNTs materials, Sun et al. designed the SCs, the anode materials of which were Fe_3_O_4_/MnO_2_/carbon materials through an electrodeposition method using a conductive yarn substrate, which showed high flexibility.^155^ It was demonstrated that areal and volumetric capacitances of 60 and 7.23 mF cm^−2^ at 0.9 mA at the 50th cycle and a capacitance retention of 65% were obtained at 3.6 mA. In addition, a discharge capacity of 127 mF cm^−2^ at 1 mA, a specific capacity of 60.74 mF cm^−2^ at 8 mA, and a high capacity retention of 48% were also achieved by the electrode. Furthermore, the SCs, which were estimated to be 10 cm, 1.5 cm^2^, and 0.0125 cm^3^ in length, area, and volume, exhibited energy densities of 0.005 mW h cm^−1^, 0.0335 mW h cm^−2^, and 4.02 mW h cm^−3^.

In conclusion, the Fe_3_O_4_/metal‐based/carbon materials are promising anode materials for practical applications in SCs, especially when the conductive substrate is applied for superb flexibility.

### 4.2 LIBs

Pure Fe_3_O_4_, Fe_3_O_4_/carbon materials, and Fe_3_O_4_/metal‐based materials (metal or metal oxide) were explored in LIBs for electrochemical performance.^121^, ^156^

#### 4.2.1 Pure Fe_3_O_4_

In addition to Fe_2_O_3_, different morphologies of bare Fe_3_O_4_, including Fe_3_O_4_ NPs,^157^, ^158^, ^159^, ^160^ Fe_3_O_4_ NWs,^161^ Fe_3_O_4_ microspheres,^162^ Fe_3_O_4_ octahedra,^163^ Fe_3_O_4_ hexahedra,^164^ hollow Fe_3_O_4_ beads,^165^ Fe_3_O_4_ thin films,^166^ and Fe_3_O_4_ nanocubes,^167^ have been studied.

**Figure 14:**
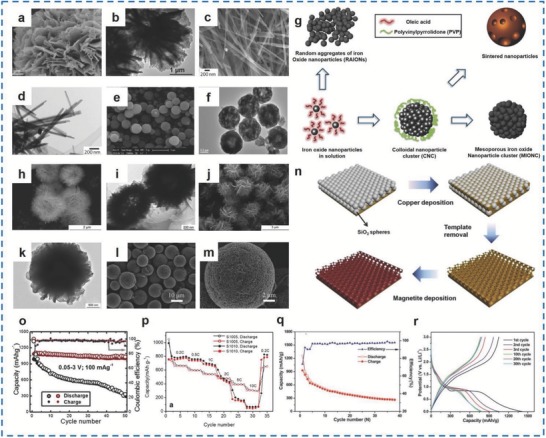
a) FESEM image of the Fe_3_O_4_ microflowers. b) TEM image of the Fe_3_O_4_ microflowers. c) Low magnification FESEM image of the as‐prepared Fe_3_O_4_. d) Low magnification TEM image of the Fe_3_O_4_ nanowires. e) Low magnification SEM image of hollow Fe_3_O_4_ spheres. f) Low magnification TEM image of hollow Fe_3_O_4_ spheres. g) Schematic illustration of the preparation of MIONCs and RAIONs (random aggregates of Fe_3_O_4_ NPs). h,j) SEM images of the flower‐like Fe_3_O_4_/carbon nanostructures. i,k) TEM images of the flower‐like Fe_3_O_4_/carbon nanostructures. l,m) FESEM images of porous Fe_3_O_4_/carbon microspheres. n) Schematic of the fabrication process for porous Fe_3_O_4_ electrodes. o) Cycling performance of Fe_3_O_4_ microflowers (red lines) and Fe_3_O_4_ microspheres (black lines) electrodes at 100 mA g^−1^ between 0.05 and 3 V. p) Electrochemical performance of Fe_3_O_4_/carbon cells: the specific capacity of S1005 (empty circles and squares) and S1010 (filled circles and squares) electrodes as a function of the cycling rate (0.2–10 C). q) Cycling performances of the Fe_3_O_4_/carbon composite. Cycling took place between 0.005 and 3.00 V versus Li/Li^+^ at a cycling rate of 924 mA g^−1^ (1 C). r) Charge–discharge profiles (1st, 2nd, 3rd, 10th, 20th, 30th cycles) of Fe_3_O_4_/carbon composite electrodes at 924 mA g^−1^ (1 C). (a,b,o) Reproduced with permission.^170^ Copyright 2016, Elsevier; (c,d) Reproduced with permission.^161^ Copyright 2013, Elsevier; (e,f) Reproduced with permission.^168^ Copyright 2012, Elsevier; (g) Reproduced with permission.^171^ Copyright 2013, American Chemical Society; (h–k) Reproduced with permission.^177^ Copyright 2014, Elsevier; (l,m) Reproduced with permission.^176^ Copyright 2016, Royal Society of Chemistry; (n) Reproduced with permission.^169^ Copyright 2014, Elsevier; (p) Reproduced with permission.^180^ Copyright 2013, Elsevier; (q,r) Reproduced with permission. Copyright 2013, Royal Society of Chemistry.^181^

Generally, strategies for preparing pure Fe_3_O_4_ mainly include hydrothermal/solvothermal method,^161^, ^168^ and the template‐electrochemical deposition method.^169^ Wang et al. prepared flower‐like bare Fe_3_O_4_ with Fe_3_O_4_ nanoplates of about 60 nm in thickness (**Figure** **14**a,b).^170^ A reversible capacity of 1000.3 mA h g^−1^ was observed over 50 cycles for the pure Fe_3_O_4_ microflowers, while the Fe_3_O_4_ microspheres only exhibited a reversible capacity of 305.3 mA h g^−1^ (Figure 14o). Su et al. prepared Fe_3_O_4_ NWs 50 nm in diameter (Figure 14c,d) via a hydrothermal method, and they displayed an initial discharge capacity of 1868 mA h g^−1^ even at 500 mA g^−1^.^161^ In addition, the pure Fe_3_O_4_ microflowers maintained a discharge capacity of 906 mA h g^−1^ even at 500 mA g^−1^. Zhang et al. fabricated Fe_3_O_4_ spheres with 400–500 nm diameters (Figure 14e,f) using a solvothermal method, and they displayed initial discharge/charge capacities of 1316 and 933 mA h g^−1^ at 500 mA g^−1^ and exhibited a coulombic efficiency of 70.9%.^168^ Wu et al. synthesized Fe_3_O_4_ electrodes with a template method based on Cu, and they deposited Fe_3_O_4_ on the template (Figure 14g).^169^ It was demonstrated that a reversible capacity of 1382 mA h g^−1^ over 100 cycles at 1 A g^−1^ was obtained for the Fe_3_O_4_ electrodes. Furthermore, initial discharge/charge capacities of 1308 mA h g^−1^ and 983 mA h g^−1^ were observed for the pure Fe_3_O_4_ with a coulombic efficiency of 75% during the 1st cycle.

Besides, a thermal decomposition approach^171^ and coprecipitation method^158^ have been applied to the fabrication of the materials. Lee et al. prepared mesoporous Fe_3_O_4_ NP (11–12 nm in size) clusters (MIONCs) by a thermal decomposition approach (Figure 14n), and they displayed a reversible capacity of 867 mA h g^−1^ during the 1st cycle.^171^ Behera et al. fabricated Fe_3_O_4_ NPs through a coprecipitation method, displaying a capacity of 1470 mA h g^−1^ for the 1st cycle and showing a reversible capacity of 1084 mA h g^−1^ at 100 mA g^−1^.^158^

#### 4.2.2 Fe_3_O_4_/Carbon Nanomaterials

Fe_3_O_4_/carbon materials can be synthesized through the hydrothermal/solvothermal–annealing/calcination method.^172^, ^173^, ^174^, ^175^ Ding et al. prepared Fe_3_O_4_/carbon microspheres, which consisted of pores with a diameter of 68 nm, carbon layers with a thickness of 21 nm, and Fe_3_O_4_ nanocrystals with 8.1 nm in size (Figure 14l,m), via a hydrothermal process.^176^ It was demonstrated that the as‐prepared materials delivered Fe_3_O_4_/carbon microspheres exhibiting a superb discharge capacity of 1231.3 mA h g^−1^ at 0.5 A g^−1^ after 100 cycles. In addition, discharge capacities of 1017.2 and 601.9 mA h g^−1^ were obtained by the Fe_3_O_4_/carbon microspheres at 1 and 5 A g^−1^ over 500 cycles, respectively. Fe_3_O_4_/carbon materials with a flower‐like structure (2 µm in diameter) (Figure 14h–k) were prepared by Deng and co‐workers via a solvothermal–calcination method in N_2_.^177^ It was demonstrated that the as‐prepared materials delivered a specific capacity of 227 mA h g^−1^ at 5 C, which was higher than that of hollow microspheres and dispersed nanoflakes with specific capacities of 45 and 10 mA h g^−1^.

Apart from above, polymerization–heat treatment process^178^, ^179^, ^180^ can also be used for the preparation of the Fe_3_O_4_/carbon materials. Jung and co‐workers fabricated Fe_3_O_4_/carbon microspheres using a polymerization–annealing two‐step process.^180^ The as‐prepared samples were divided into three different kinds, namely, S1001, S1005, and S1010. Among them, the S1001 had the largest average diameter because the Fe_3_O_4_ NPs/CA in this sample was scarce. Furthermore, S1005 was smoother than S1010. A discharge capacity of 1225 mA h g^−1^ was obtained by S1010 for the 1st cycle (Figure 14p). In addition, Wu et al. synthesized Fe_3_O_4_/carbon materials by mixing Fe(NO_3_)_3_, citric acid, and NaCl in the solution and drying them to form a citric acid network, which was embedded with Fe nitrate and NaCl.^181^ As a result, the Fe_3_O_4_/carbon materials exhibited a reversible capacity of over 780 mA h g^−1^ over 20 cycles and 834 mA h g^−1^ over 60 cycles (Figure 14q). Furthermore, the as‐prepared materials displayed discharge/charge capacities of 1415 and 1050 mA h g^−1^, while the Fe_3_O_4_ NPs only exhibited 1262 and 929 mA h g^−1^ (Figure 14r).

**Figure 15:**
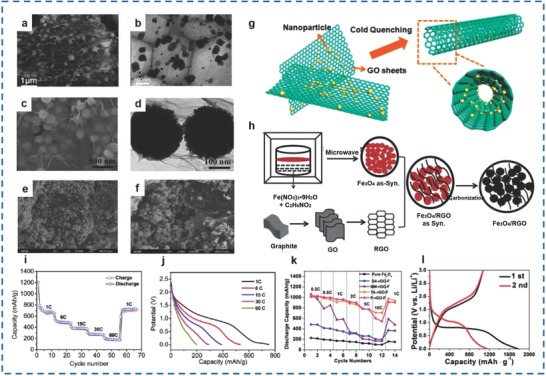
a) SEM image of the cross‐section of GNS/Fe_3_O_4_ composite. b) TEM image of GNS/Fe_3_O_4_ composite. c,d) SEM and TEM images of Fe_3_O_4_/graphene framework. e,f) SEM images of Fe_3_O_4_/rGO composite. g) Schematic diagram of synthetic route of Fe_3_O_4_/GNSs composite. h) Schematic illustration of the synthesis process of Fe_3_O_4_/rGO composite. i) Rate capability of the Fe_3_O_4_/graphene electrode at different discharge and charge rates. j) Discharge–charge voltage‐capacity profiles of the Fe_3_O_4_/GF electrode at different current rates. k) Cycling performance at 1 C of the pure Fe_3_O_4_, self‐assembled rGO/Fe_3_O_4_ hybrid paper (SA‐rGO‐F), the mechanical mixture of rGO and Fe_3_O_4_ (MM‐rGO‐F), thermally annealed rGO/Fe_3_O_4_ hybrid paper (TA‐rGO‐F), and powdered rGO/Fe_3_O_4_ (P‐rGO‐F). l) Charge–discharge voltage profiles at 1 C of the TA‐rGO‐F. (a,b) Reproduced with permission.^188^ Copyright 2010, American Chemical Society; (c,d) Reproduced with permission.^191^ Copyright 2015, Royal Society of Chemistry; (e,f) Reproduced with permission.^183^ Copyright 2015, Royal Society of Chemistry; (g) Reproduced with permission.^189^ Copyright 2014, American Chemical Society; (h) Reproduced with permission.^173^ Copyright 2014, Royal Society of Chemistry; (i,j) Reproduced with permission.^190^ Copyright 2013, American Chemical Society; (k,l) Reproduced with permission.^194^ Copyright 2014, Royal Society of Chemistry.

Fe_3_O_4_ nanocrystals/GNSs,^182^ Fe_3_O_4_/graphene hollow spheres,^183^ Fe_3_O_4_ NR/graphene materials,^174^, ^184^ hollow Fe_3_O_4_/graphene films,^185^ Fe_3_O_4_ micropheres decorated on GNSs,^186^ and Fe_3_O_4_ NPs wrapped by graphene nanoscrolls^187^ have also been explored. Zhou et al. anchored Fe_3_O_4_ particles on GNSs (**Figure** **15**a,b) using an in situ reduction method, and the size of the Fe_3_O_4_ particles decreased to 428 nm over 30 cycling tests.^188^ It was demonstrated that Fe_3_O_4_/GNS materials displayed a charge capacity of 900 mA h g^−1^, while commercial Fe_3_O_4_ particles only exhibited 770 mA h g^−1^ for the 1st cycle. Furthermore, a specific capacity of 520 mA h g^−1^ at 1750 mA g^−1^, which was 53% of the initial capacity, was also obtained for the as‐prepared materials. Zhao et al. fabricated Fe_3_O_4_/GNSs using a preparation method (Figure 15g).^189^ The initial discharge/charge capacities of Fe_3_O_4_/GNSs and Fe_3_O_4_/GSs were ≈1720 and 1410 mA h g^−1^, and 1060 and 960 mA h g^−1^ at 0.1 C, respectively. Luo et al. synthesized the Fe_3_O_4_/graphene materials via an atomic layer deposition method, which delivered an initial capacity of 1192 mA h g^−1^, which decreased to 785 mA h g^−1^ for the 2nd cycle at 1 C, leading to a coulombic efficiency of ≈66%.^190^ In addition, the as‐prepared materials exhibited a discharge capacity of 785 mA h g^−1^ at 1 C and retained that capacity for 500 cycles (Figure 15i,j). Ren et al. prepared Fe_3_O_4_/graphene materials via a hydrothermal method (Figure 15c,d).^191^ As a result, the Fe_3_O_4_/graphene materials displayed a superb reversible capacity of 1164 mA h g^−1^ after 500 cycling tests at 500 mA g^−1^.

Fe_3_O_4_/rGO materials can be fabricated by a solvothermal method,^192^ deposition method,^193^ and ultrasonication–heat treatment technology.^194^ Chen and co‐workers synthesized Fe_3_O_4_/rGO materials with Fe_3_O_4_ particles (160 nm in diameter) using a solvothermal method (Figure 15e,f), and initial discharge/charge capacities of 1912 and 1450 mA h g^−1^ were achieved by the as‐synthesized materials, while those of bare Fe_3_O_4_ were only 1342 and 991 mA h g^−1^ at 92.4 mA g^−1^.^192^ Furthermore, Fe_3_O_4_/rGO materials maintained a capacity of 1547 mA h g^−1^, which was higher than that of pure Fe_3_O_4_ 775 mA h g^−1^ during the 5th cycle. In addition, the materials also exhibited a superb reversible capacity of 1031 mA h g^−1^ over 50 cycles, which was 84% of the initial value. Bhuvaneswari et al. synthesized Fe_3_O_4_/rGO materials by decorating Fe_3_O_4_ particles on rGO (Figure 15h), which displayed specific capacities of ≈612 and ≈446 mA h g^−1^ at 1 and 5 C, respectively.^193^ Zhang et al. fabricated Fe_3_O_4_ nanocrystal/rGO papers (about 2 nm) via an ultrasonication–heat treatment technology, which exhibited a superb reversible capacity of 1140 mA h g^−1^ at 1 C.^194^ It was demonstrated that the mechanical mixture of rGO and Fe_3_O_4_ (MM‐rGO‐F) delivered a specific capacity of 150 mA h g^−1^ while the thermally annealed Fe_3_O_4_/rGO hybrid papers (TA‐rGO‐F) showed a reversible capacity of ≈1140 mA h g^−1^ for 220th cycle and the powdered rGO/Fe_3_O_4_ (P‐rGO‐F) displayed the first reversible capacity of 1521 mA h g^−1^ (Figure 15k,l).

Generally, Fe_3_O_4_/carbon/graphene materials can be synthesized by a hydrothermal/ solvothermal method.^195^, ^196^, ^197^ Zuo et al. fabricated homogeneously anchored H‐Fe_3_O_4_ NPs (100–150 nm in diameter) on graphene nanosheets (H‐Fe_3_O_4_/carbon/GNSs) via a hydrothermal method, which exhibited an initial discharge capacity of 1331.7 mA h g^−1^ at 0.1 C, while those of pure Fe_3_O_4_ and graphene were only 926 and 744 mA h g^−1^, respectively.^196^ Similarly, the hydrothermal method was also used by Fan and co‐workers for the fabrication of Fe_3_O_4_/carbon/graphene materials by decorating GNSs with Fe_3_O_4_/carbon NPs 50 nm in size.^197^ It was demonstrated that the as‐fabricated Fe_3_O_4_/carbon/graphene materials displayed initial reversible capacities of 1016.6 and 1199.8 mA h g^−1^ over 500 cycling tests at 1.0 A g^−1^ and exhibited a retention of 52.8% at 0.2 A g^−1^. Zhang et al. synthesized graphene/Fe_3_O_4_/carbon materials with core–shell nanosheet structures with Fe_3_O_4_ NPs of 6 nm in size, and they showed superior electrochemical performance with a good reversible capacity of 1468 mA h g^−1^ and a superb cycle stability of 1200 mA h g^−1^ over 100 cycling materials at 0.2 A g^−1^.^195^ Zhao et al. prepared Fe_3_O_4_/carbon/graphene materials with a sandwich morphology and found that the as‐prepared materials displayed capacities of ≈1481 mA h g^−1^ for the 1st cycle and ≈860 mA h g^−1^ over 100 cycles at 0.1 C.^198^ Hence, the Fe_3_O_4_/carbon/graphene materials are superb anode materials for high performance in LIBs.

#### 4.2.3 Fe_2_O_3_/Metal‐Based Nanomaterials

Fe_2_O_3_/metal‐based materials, which include Fe_3_O_4_/Cu NWs,^199^ Fe_3_O_4_/Ni films,^200^ Fe_3_O_4_/γ‐Fe_2_O_3_ microspheres,^201^ Fe_3_O_4_/CuO NWs^202^ and Fe_3_O_4_ NPs dropped on TiO_2_ nanofibers^203^ have also been tested as anodes for LIBs.

**Figure 16:**
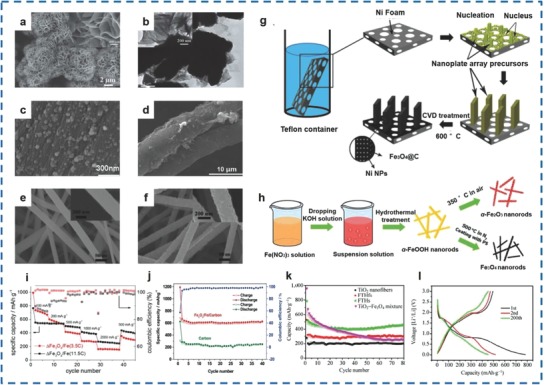
a,b) SEM and TEM images (inset: magnified SEM image) of the 3D porous nano‐Ni/Fe_3_O_4_ composite. c) SEM image of Fe_3_O_4_/Fe/MBCFs after the calcinations. d) SEM image of Fe_3_O_4_/Fe/MBCFs after 50 charging cycles at 2 A g^−1^, showing the intact fibrous geometry with appearance of rough surface. e,f) Low and high resolution (inset) SEM images of bare TiO_2_ nanofibers and FTHs (TiO_2_ /Fe_3_O_4_ with a few secondary Fe_3_O_4_ NPs). g) Schematic mechanism for the direct‐growth process of Fe_3_O_4_/Ni/caebon nanoplate arrays on Ni foam. h) Schematic representation of synthesis of single‐crystalline mesoporous α‐Fe_3_O_4_ NRs and Fe_3_O_4_ NRs. i) Variable current rate tests for samples Fe_3_O_4_/Fe (3.5 C) and Fe_3_O_4_/Fe (11.5 C). j) Cycling stability of Fe_3_O_4_/Fe/carbon composite electrode. k) Cycling performance of bare TiO_2_ nanofibers, FTHs (TiO_2_ /Fe_3_O_4_ nanofibers with a few secondary Fe_3_O_4_ NPs), FTHs, and TiO_2_ –Fe_3_O_4_ physical mixture(70 wt% of active materials) at 100 mA g^−1^. l) Discharge and charge curves of FTHs at 100 mA g^−1^. (a,b) Reproduced with permission.^200^ Copyright 2012, Royal Society of Chemistry; (c,d) Reproduced with permission.^207^ Copyright 2015, Elsevier; (e,f,k,l) Reproduced with permission.^203^ Copyright 2015; (g) Reproduced with permission.^205^ Copyright 2015, Elsevier; (h) Reproduced with permission.^208^ Copyright 2012; (i) Reproduced with permission.^204^ Copyright 2015, Elsevier; (j) Reproduced with permission.^206^ Copyright 2012, American Chemical Society.

Fe_3_O_4_/Fe materials were synthesized by Lübke et al. using a hydrothermal method and were tested as anode materials for LIBs; they delivered a good capacity of 390 mA h g^−1^ over 50 cycles at 200 mA g^−1^ (**Figure** **16**i).^204^ Similarly, a hydrothermal–heat treatment method was also used by Li et al. for the fabrication of Fe_3_O_4_/Ni/carbon nanoplate arrays (Figure 16g).^205^ The as‐prepared materials showed discharge capacities of 832.5 and 279 mA h g^−1^ at 0.3 and 4.5 C over 50 cycles, respectively. In addition, the materials maintained a specific capacity of 279 mA h g^−1^ at 4.5 C. Furthermore, Wang et al. decorated Fe_3_O_4_/NPs on TiO_2_ nanofiber (≈220 nm in diameter) hierarchical heterostructures (FTHs) by combining the electrospinning and hydrothermal methods together (Figure 16e,f).^203^ The initial discharge/charge capacities of 783.6 and 494.5 mA h g^−1^ were achieved for the as‐synthesized materials at 100 mA g^−1^. In addition, the materials even showed a reversible capacity of 454.5 mA h g^−1^ over 200 cycles (Figure 16k,l).

Apart from the hydrothermal method, the electrochemical plating method^200^ and sol–gel polymerization–heat‐treatment method^206^ have also been used for the synthesis of Ni/Fe_3_O_4_ films and Fe_3_O_4_/Fe/carbon materials. Xiong et al. prepared porous Fe_3_O_4_/Ni films (the Ni film was 200–300 nm in diameter) with flower structures via an electrochemical plating method (Figure 16a,b).^200^ The initial discharge/charge capacities of 1324.3 and 1138.6 mA h g^−1^ at 0.1 C were achieved for the as‐synthesized materials, and they were higher compared with the value for the pure Fe_3_O_4_ film (873.7 and 582.6 mA h g^−1^). In addition, the initial coulombic efficiency of the Fe_3_O_4_/Ni films was 86.0%, whereas that of the Fe_3_O_4_ films was 66.7%. Hence, the materials displayed an outstanding cycle stability (951.9 mA h g^−1^ at 1 C over 50 cycles) and a superior rate capability (772.1 mA h g^−1^ at 5 C). The sol–gel polymerization and heat‐treatment methods were used by Zhao and co‐workers for the fabrication of Fe_3_O_4_/Fe/carbon materials (Figure 16j).^206^ The Fe_3_O_4_/Fe/carbon electrodes exhibited initial discharge/charge capacities of 1192 and 685 mA h g^−1^ at 50 mA g^−1^, while the specific capacities of the Fe_3_O_4_ and carbon were only 926 and 372 mA h g^−1^. Zhang et al. prepared mesoporous biocarbon fibers (Fe_3_O_4_/Fe/MBCFs) by using a natural cotton biotemplate (Figure 16c,d).^207^ The Fe_3_O_4_/Fe/MBCFs exhibited an initial reversible capacity of 1340.5 mA h g^−1^ at 1 A g^−1^, a reversible capacity of 533.2 mA h g^−1^ in the 10th cycle and maintained 524.6 mA h g^−1^ after 60 cycling tests. In summary, Fe_3_O_4_‐MO*_x_* are superb candidates for high electrochemical performance in energy storage devices.

A Fe_3_O_4_ nanoflake/N‐doped carbon matrix (Fe_3_O_4_ NFs/NC),^209^ N‐doped carbon wrapped Fe_3_O_4_ (N‐mFe_3_O_4_/carbon) nanospheres,^210^ N‐doped Fe_3_O_4_/carbon materials with an urchin structure,^211^ and Fe_3_O_4_/N‐carbon (Fe_3_O_4_/CN) core–shell microspheres^212^ have been explored.

N‐doped Fe_3_O_4_/carbon materials are mainly synthesized via hydrothermal/solvothermal–annealing method.^209^ The N‐doped Fe_3_O_4_/carbon materials with an urchin morphology (500 nm–1 µm) were prepared by Chen and co‐workers via a hydrothermal–carbonization method, and as a result, a reversible capacity of 800 mA h g^−1^ over 100 cycles at 500 mA g^−1^ was obtained for the materials.^211^ Guo et al. fabricated Fe_3_O_4_ NF/NC with Fe_3_O_4_ NF (50–60 nm in width) and NC (10 nm in thickness).^209^ The as‐prepared materials (proportion of carbon is 44%) displayed reversible capacities of 1046 mA h g^−1^ at 0.2 C after 200 cycles, 662 mA h g^−1^ at 1 C over 500 cycles, and 600 mA h g^−1^ at 5 C after 200 cycles, showing superior performance in LIBs. Similarly, Meng et al. prepared N‐mFe_3_O_4_/carbon materials with Fe_3_O_4_ particles (≈115 nm in size) and a carbon coating (2–5 nm in thickness), and they displayed a reversible capacity of 1273 mA h g^−1^ after 200 cycles at 0.2 C and showed an outstanding rate capability of 596 mA h g^−1^ at 1 C and 441 mA h g^−1^at 2 C.^210^ Wang et al. fabricated Fe_3_O_4_/CN core–shell microspheres with a peanut structure using a solvothermal method, and they displayed a reversible capacity of 670 mA h g^−1^ at 0.01–3.0 V over 30 cycles and were higher than that of pure Fe_3_O_4_ (100 mA h g^−1^).^212^ Hence, the N‐doped Fe_3_O_4_/carbon materials are proved to be superb candidates in LIBs.

### 4.3 SIBs

Fe_3_O_4_ NPs,^213^ octahedral Fe_3_O_4_/carbon materials,^214^ and Fe_3_O_4_ quantum dots implanted in microcarbon/graphene (N source) materials (Fe_3_O_4_ QD/C‐GN)^215^ have been explored in SIBs for superb electrochemical performance. Pure Fe_3_O_4_ NPs (<10 nm) were fabricated by Kumar and co‐workers via a hydrothermal method, and they exhibited an initial discharge capacity of 590 mA h g^−1^ and a reversible capacity of 248 mA h g^−1^ over 50 cycles at 0.1 C.^213^ In addition, Li et al. synthesized the Fe_3_O_4_/carbon nano‐octahedra with 300–500 nm diameters through a pyrolysis method. It was demonstrated that initial discharge/charge capacities of 824.5 and 496.2 mA h g^−1^, 663.6 and 358.0 mA h g^−1^ were achieved by the as‐obtained materials at 100 and 500 mA g^−1^.^214^ In addition, the Fe_3_O_4_/carbon materials also exhibited coulombic efficiencies of 60.2% and 53.9% for the 1st cycle at 100 and 500 mA g^−1^, respectively. Qi et al. obtained the Fe_3_O_4_ QD/C‐GN materials on a substrate of metal organic frameworks using an in situ quantization method.^215^ The as‐prepared materials showed initial discharge/charge capacities of 1081 and 971 mA h g^−1^ and a coulombic efficiency of 62.4% at 200 mA g^−1^, which were higher compared to the values for bulk Fe_3_O_4_‐GN (680, 461 mA h g^−1^, 44.5%). In summary, pure Fe_3_O_4_, Fe_3_O_4_/carbon materials and Fe_3_O_4_ QD/C‐GN materials all exhibit great potential for applications in energy storage devices.

### 4.4 Others

Fe_3_O_4_ or Fe_3_O_4_‐based materials are also applied in other batteries including LSBs, alkaline secondary batteries, and Fe/air batteries. Fe_3_O_4_ NP/graphene materials with a particle size of 40–60 nm for LSBs were obtained by Fu et al. through a coprecipitation method.^216^ As a result, a specific capacity of 1430 mA h g^−1^ over 100 cycling tests at 200 mA g^−1^ was reached by the as‐fabricated materials. Furthermore, the Fe_3_O_4_ NPs/graphene materials exhibited discharge capacities of 855 and ≈210 mA h g^−1^ for the 1st cycle and over 40 cycles, respectively. Similarly, a coprecipitation method was also used by Li et al. for the synthesis of pure Fe_3_O_4_ in alkaline secondary batteries.^17^ The as‐prepared materials annealed at 700 °C performed best, delivering discharge capacities of 587.6, 539.5, and 500.1 mA h g^−1^ at 240, 600, and 1200 mA g^−1^, respectively. In addition, Ito et al. fabricated Fe_3_O_4_/tubular carbon nanofibers (the CNFs were 50 nm in diameter) (Fe_3_O_4_/TCNFs) for Fe/air batteries, and they displayed a good capacity of 786 mA h g^−1^ and a cycling efficiency of 76% after 30 cycles.^217^

A brief comparison of the synthesis methods and electrochemical performances of the Fe_3_O_4_‐based materials for rechargeable batteries is shown in **Table** **3**.

**Table 3:** Fe_3_O_4_‐based materials as anodes for rechargeable batteries

Materials	Methods	CDa) [C or mA g^−1^] DC/CCb) [mA h g^−1^]	Capacity [mA h g^−1^]/Cycles
**LIBs**			
Fe_3_O_4_ nanowires^161^	Hydrothermal, annealing	500,1868,–	503,100
Fe_3_O_4_ 170	Solvothermal method	100,1365.4,1084.1	1000.3,50
Fe_3_O_4_ NPs@C^218^	Sucrose calcination, annealing	924,–,–	773,200
Fe_3_O_4_ particles@C^219^	Self‐assembly, syn‐carbonization	100,–,–	932,100
Fe_3_O_4_ nanocrystals@CNT^220^	Coprecipitation, sonication, oil bath	C/0.1,–,–	850,100
Fe_3_O_4_@C composite nanofibers^221^	Electrospinning, carbonization	200,1551,–	1000,80
Fe_3_O_4_@G films^222^	Solvothermal method, annealing	100,–,–	1038,100
Fe_3_O_4_ NPs@rGO^223^	Coprecipitation, reduction	1000,–,–	300,100
Fe_3_O_4_@C@G^197^	Hydrothermal, annealing	200,1450.7,1016.6	633.5,500
Fe_3_O_4_@Fe@C^206^	Sol–gel polymerization, heat‐treatment	50,1192,685	600,40
Fe_3_O_4_@Fe_2_O_3_ NRs^208^	Hydrothermal, calcination	C/0.1,1230,4,955.8	893.3,50
Fe_3_O_4_@α‐Fe_2_O_3_@C NRs^224^	Hydrothermal, annealing	C/0.05,2008,–	435,50
Fe_3_O_4_@N@C^211^	Hydrothermal, carbonization	500,–,–	800,100
**SIBs**			
Fe_3_O_4_ NPs^213^	Hydrothermal, annealing	83,590,–	248,50
Fe_3_O_4_@C nanooctahedra^214^	Pyrolysis	100,643,–	380,60
Fe_3_O_4_@graphene sheets^225^	Hummers method	200,1081, 971	343,200
**LSBs**			
Fe_3_O_4_ NPs@G^216^	Coprecipitation, annealing	200,2670,–	1430,100

^a)^ CD: Current density (C or mA g^−1^)

^b)^ DC/CC: Initial discharge/charge capacity (mA h g^−1^).

## 5 FeO‐Based Nanomaterials

FeO is black power without magnetism which can be applied in electrochemical energy storage, including SCs^226^ and LIBs.^227^, ^228^, ^229^ A method which using oleic acid (OA) and oleylamine (OAm) to react with iron(III) acetylacetonate ([Fe(acac)_3_]) at high temperature to synthesize the FeO NPs was reported by Hou et al.^230^ In fact, pure FeO NPs are difficult to prepare because they were easy to be oxidized to Fe_2_O_3_ and Fe_3_O_4_ owing to the chemically unstable structure. Hydrothermal method was used for the synthesis of FeO/CVO with FeO nanospheres and CVO (cobalt vanadium oxide hydrate) in SCs by Centre et al.^226^ It was demonstrated that a specific capacitance of 968 mA h g^−1^ was achieved at 1 A g^−1^ and a capacitance retention of 95% was achieved after 5000 cycles.

FeO/C,^227^ Fe_3_O_4_/FeO/Fe,^228^ Fe_3_O_4_/FeO/Fe/C^229^ have been investigated as anode materials for LIBs, respectively. Carbothermally reduction method, soild method, and hydrothermal‐treatment method can be used to synthesize them. Gao et al. synthesized the FeO/C anode materials from nano Fe_2_O_3_ combined with acetylene black (AB) via a carbothermal reduction method.^227^ It was demonstrated that the initial discharge/charge capacities of FeO/C composites with carbon ratio of 30, 40, 50, 60, 70 wt% are 653, 760, 719 645, 630 mA h g^−1^ at 1 C, respectively. So, the FeO/C (40 wt%) materials performed better with a coulombic efficiency of 74% at the 1st cycle. Research on the Fe_3_O_4_/FeO/Fe was done by Shi et al. and they found that an initial discharge capacity and a coulombic efficiency of 286.5 mA h g^−1^ and 90.8% was achieved at 5 C.^228^ Zhao et al. prepared the Fe_3_O_4_/FeO/Fe/C materials by a hydrothermal‐treatment method without any templates.^229^ The discharge capacities of 1722 and 550 mA h g^−1^ were achieved in the 1st and 60th at 88 mA g^−1^, respectively.

In general, synthesis for composites which combine FeO with Fe_2_O_3_ or Fe_3_O_4_ is easy while that of pure FeO is complex and difficult. The controlled oxidation of the FeO NPs will result in the formation of Fe_2_O_3_ or Fe_3_O_4_ NPs. So, when the reversible capacity and rate capability are tested at different temperatures, the component of the composites may change. That is why there are very few applications of FeO in electrochemical energy storage.

## 6 Conclusions and Outlook

Currently, FeO*_x_* have been paid more attention because of the good theoretical capacity, rich abundance, low cost, and environmental friendliness. Apart from the advantages, they also have the drawbacks of poor conductivity and unstable structures. To overcome the problems, considerable efforts have been done by the scientists for better electrochemical performances. This review provides an overview of FeO*_x_* (Fe_2_O_3_/Fe_3_O_4_)‐based materials for applications in energy storage devices, including SCs, LIBs, SIBs, and other batteries.^231^, ^232^ The synthetic methods, morphologies, and electrochemical performance are mainly introduced. The combination of different materials with Fe_2_O_3_/Fe_3_O_4_ achieved electrochemical properties of high capacity, good rate capability, and cycling life.

It is crucial to improve the undesirable electrochemical and cycling performance of FeO*_x_* caused by the poor conductivity and unstable structures. Three kinds of materials, including carbon‐based materials (amorphous carbon, graphene, CNTs, and so on), conductive polymers (polymer polypyrrole (PPy), polyaniline (PANI), polythiophenes (PTh), and poly(3,4‐ethylenedioxythiophene (PEDOT)), and metal materials (metal NPs and metal substrates such as Ni/Cu foam) have been used combined with FeO*_x_* for enhanced conductivity. Besides, desirable structures are designed for large surface areas and good mechanical and chemical abilities, including nanowires, nanorods, nanosheets, nanotubes, and so on, to accelerate the transfer of the charge and shorten the ion diffusion pathway.

Apart from the advanced achievements, challenges and bottleneck still exist: 1) The crystal structures may have some effects on electrochemical performance so investigation of this aspect should be focused. 2) The interaction force between the combination of FeO*_x_* and other different materials may interfere with the charge transfer and more research should be done to investigate the mechanism in this respect and avoid the disadvantages. 3) The synthesis for pure FeO is difficult and the components of FeO‐based materials are changeable so that the research on the electrochemical performances is lacking, which causes the shortage of applications for electrochemical energy storage. To achieve more desired performance characteristics, future directions in the study of applications for FeO*_x_*‐based materials for energy storage are as follows: 1) Unique nanostructures and porous structures with large surface areas are required for improving the transport and activities of the electrodes. 2) Highly conductive substrates should be utilized for the synthesis of integrated electrodes. The FeO*_x_* materials grown on the substrates with nanowire, nanotube, and nanorod morphologies possess good conductivity and superb cycle stability. 3) Flexible energy storage devices based on FeO*_x_*‐based materials should be more widely studied for practical applications.^233^ 4) Considerable efforts should be made in componential regulation, elemental doping, and defect modification for better electrochemical performance. 5) Due to the advantages of surface modification on electrodes, development of new active materials, and design of optimized nanostructures, ALD method should be paid more attention for the improved rate capability and cycling stability. In summary, rapid progress has been achieved for FeO*_x_*‐based materials resulting in good electrochemical performance, and FeO*_x_*‐based materials are expected to have broad applications in electrochemical energy storage.

## Conflict of Interest

The authors declare no conflict of interest.
